# Intrinsic geometric super-twisting control on Lie groups with application to spacecraft attitude tracking

**DOI:** 10.3389/frobt.2026.1961299

**Published:** 2026-10-28

**Authors:** Pierantonio Bertuccio, Mauro Mancini, Elisa Capello

**Affiliations:** Politecnico di Torino, Department of Aerospace and Mechanical Engineering, Turin, Italy

**Keywords:** sliding-mode control, higher-order sliding modes, super-twisting algorithm, Lie groups, geometric control, spacecraft attitude control, disturbance rejection

## Abstract

Spacecraft control requires globally consistent formulations and robustness against model uncertainties and external disturbances. While a general intrinsic sliding-mode framework on Lie groups is available for first-order reaching, existing higher-order geometric sliding-mode formulations are predominantly developed for specific configuration groups or application models. This paper addresses the complementary problem of formulating a multivariable Super-Twisting reaching mechanism directly within a general Lie-group mechanical framework. The algorithm is formulated on the Lie algebra through covariant derivatives and builds upon the existing state-space Lie group structure. A strict Lyapunov analysis establishes finite-time convergence to the second-order sliding set and provides constructive gain conditions. Under suitable matching and regularity assumptions, exact rejection of matched disturbances is obtained, while the sliding dynamics ensure almost-global asymptotic tracking. The method is specialized to spacecraft attitude dynamics on SO(3) and assessed under nominal, disturbed, uncertain, and sampled-data conditions.

## 1 Introduction

Spacecraft attitude control requires accurate large-angle rotational maneuvers in the presence of model uncertainty, environmental disturbances, and actuator limitations (Schaub and Junkins, 2018; Zhang and Duan, 2023). The choice of attitude representation plays an important role in the resulting control formulation. Minimal parameterizations such as Euler angles introduce kinematic singularities, whereas unit quaternions provide a nonsingular representation at the price of redundancy and a double covering of SO(3) , which may lead to unwinding unless suitable control mechanisms are introduced (Wen and Kreutz-Delgado, 1991; Chaturvedi et al., 2011; Bhat and Bernstein, 2000; Mayhew et al., 2011). Rotation matrices avoid both local singularities and quaternion ambiguity, but evolve on the nonlinear manifold SO(3) . These considerations motivate control formulations that explicitly account for the geometry of the underlying configuration space while also ensuring robustness against model uncertainty and external disturbances.

Geometric control directly addresses the geometric aspect by formulating the dynamics and control laws on the configuration manifold. The rotational configuration of a rigid spacecraft naturally evolves on SO(3) , whereas combined translational and rotational motion can be described on SE(3) . Intrinsic formulations preserve the nonlinear structure of these spaces and avoid the introduction of auxiliary local attitude coordinates (Bullo and Murray, 1995; 1999; Chaturvedi et al., 2011; Lee, 2011; Lee et al., 2010). Owing to the topology of SO(3) , however, continuous time-invariant feedback cannot provide global asymptotic stabilization of a single attitude equilibrium, and smooth geometric controllers generally establish almost-global convergence (Bhat and Bernstein, 2000). More importantly for the present work, geometric consistency alone does not provide robustness against model uncertainty and external disturbances.

Sliding-mode control (SMC) is particularly attractive in this respect because it drives the system trajectories toward a prescribed sliding set and provides robustness with respect to matched perturbations (Utkin, 1992). Geometric SMC formulations have therefore been developed directly on nonlinear configuration spaces. Espindola and Tang (2025) introduced a general intrinsic sliding-mode framework for fully actuated mechanical systems evolving on Lie groups, which is particularly relevant to the present work. Their construction equips the tracking-error state space with a Lie-group structure and defines a corresponding sliding subgroup, thereby providing a systematic geometric description of the sliding motion. The state-space Lie group and sliding-subgroup construction introduced in (Espindola and Tang, 2025) is adopted here as the geometric foundation of the present work. However, their controller employs a first-order reaching mechanism to drive the system trajectories toward the sliding subgroup.

The reaching mechanism of conventional first-order SMC is nevertheless based on a discontinuous control action. In practical aerospace implementations, the associated high-frequency switching may interact with finite sampling rates, actuator bandwidth, sensor noise, flexible dynamics, and other neglected effects, producing undesirable oscillatory behavior. As discussed by Levant (2010), the chattering observed in an implemented sliding-mode controller depends not only on the ideal continuous-time reaching law but also on the dynamics and discretization of the implementation. Higher-order sliding-mode methods address the discontinuity of first-order SMC by transferring it to a higher-order derivative or to an internal controller state while retaining finite-time sliding properties (Levant, 1998). Among them, the Super-Twisting Algorithm (STA) is particularly relevant because, under appropriate regularity assumptions, it establishes a second-order sliding regime while producing a continuous ideal control input without requiring explicit knowledge of the sliding-variable derivative (Levant, 1993). This continuity property is inherent to the Super-Twisting mechanism and is therefore not a distinctive feature of the geometric formulation proposed in this work.

The theoretical foundations of the STA are well established in Euclidean spaces. Strict Lyapunov constructions for the classical STA provide finite-time convergence conditions and reaching-time estimates (Moreno and Osorio, 2008; 2012), while multivariable extensions have addressed coupled Euclidean systems, matched perturbations, and uncertain input matrices (Moreno et al., 2022). Higher-order sliding-mode and Super-Twisting concepts have also been combined with geometric control in several application-specific settings. Meng et al. (2023) developed a second-order sliding-mode controller specifically for spacecraft attitude dynamics on SO(3) , with finite-time reaching established through a homogeneity-based analysis. Krishna et al. (2021) considered a multivariable Super-Twisting-based geometric attitude-control design for a rotorcraft, while Krishna and Kothari (2022) developed a robust geometric trajectory-tracking controller for a variable-pitch quadrotor in which bounded disturbances and parametric uncertainties are handled through a Super-Twisting Algorithm. Gong et al. (2024) developed an adaptive generalized Super-Twisting controller for fixed-time spacecraft pose tracking on SE(3) , with the tracking error represented through exponential coordinates. These contributions extend higher-order sliding-mode concepts to several Lie-group-based aerospace control problems. However, the resulting formulations remain tailored to specific configuration groups or application models and, in the case of (Gong et al., 2024), employ an exponential-coordinate representation of the configuration error.

In parallel, recent spacecraft and aerospace sliding-mode research has addressed complementary design issues. Das and Sinha (2023) developed an unwinding-free fast finite-time spacecraft attitude-tracking controller based on modified Rodrigues parameters. Wang et al. (2025) proposed a quaternion-based data-driven adaptive attitude controller combined with a globally nonsingular fast terminal sliding-mode law in the presence of inertia uncertainty. Actuator saturation and finite-time reaching for spacecraft attitude control using modified Rodrigues parameters were considered by Guo et al. (2023), while Sun et al. (2025) addressed three-dimensional maneuver control of a spinning tether system under limited thrust authority through a Lagrangian sliding-mode formulation. These developments address important issues including finite-time convergence, unwinding avoidance, nonsingular formulations, and actuator constraints, but pursue different design objectives from the general intrinsic higher-order construction considered here.

The literature discussed above points to two complementary lines of development. General intrinsic sliding-mode frameworks are available for fully actuated mechanical systems on Lie groups (Espindola and Tang, 2025), while multivariable Super-Twisting theory is well established in Euclidean spaces (Moreno et al., 2022). Higher-order geometric sliding-mode controllers have also been developed for specific configuration groups, such as SO(3) (Meng et al., 2023). Against this background, the present work extends the general intrinsic sliding-mode framework by formulating a multivariable Super-Twisting mechanism directly on the Lie algebra. The formulation preserves the geometric structure of the mechanical system without introducing an auxiliary Euclidean parametrization of the configuration manifold, while enabling a rigorous finite-time stability analysis.

More specifically, the problem addressed in this work is to construct an intrinsic multivariable Super-Twisting reaching mechanism within the general geometric sliding-mode framework introduced by Espindola and Tang (2025), while preserving its Lie-group formulation. To this end, both layers of the proposed reaching dynamics are formulated on the Lie algebra through covariant derivatives associated with the mechanical metric. A strict metric Lyapunov construction, inspired by the Euclidean Super-Twisting analysis of Moreno and Osorio 2008, 2012, is then developed to establish finite-time reaching, constructive nominal and robust gain conditions, explicit reaching-time bounds, and finite-time compensation of sufficiently regular matched disturbances.

The resulting framework is general, whereas rigid-spacecraft attitude tracking on SO(3) is considered as the principal application. The general construction is specialized to the spacecraft rotational dynamics to obtain an explicit torque-control law, which is then assessed against first-order geometric sliding-mode baselines under matched disturbances, model uncertainty, actuator imperfections, and sampled-data implementation. The intrinsic formulation does not remove the topological limitations of SO(3) , and the resulting spacecraft tracking property therefore remains almost global.

The specific contributions of this work can be summarized as follows.

- An intrinsic multivariable Super-Twisting reaching law is formulated for fully actuated mechanical systems evolving on general Lie groups, with both controller layers expressed directly on the Lie algebra through the Levi–Civita covariant derivative.
- A strict Lyapunov analysis, formulated covariantly through the mechanical metric and the associated Levi–Civita connection, establishes finite-time convergence to the second-order sliding set, provides constructive nominal and robust gain conditions and explicit reaching-time bounds, and proves finite-time compensation of matched disturbances satisfying the stated regularity assumptions.
- The general framework is specialized to rigid-spacecraft attitude tracking on SO(3) and evaluated against first-order geometric sliding-mode controllers through comparative, Monte Carlo robustness, and gain-sensitivity simulations under sampled-data and actuator-imperfect conditions.

A compact summary of the main theoretical distinctions between the present contribution and the closest related approaches is provided in Table 1.

**TABLE 1:** Positioning of the present work with respect to the closest geometric and super-twisting formulations.

Work	Configuration	Main analytical positioning	Error representation	Reaching law	Main distinction
(Meng et al., 2023)	SO(3)	Spacecraft-attitude-specific second-order sliding-mode formulation with homogeneity-based finite-time reaching analysis	SO(3) -specific geometric attitude and velocity errors	Second-order SMC	Higher-order geometric sliding-mode design specialized to spacecraft attitude dynamics on SO(3)
(Gong et al., 2024)	SE(3)	Adaptive fixed-time spacecraft pose-control design with dual-layer gain adaptation	Exponential coordinates of the SE(3) pose-tracking error	Adaptive generalized STA	Higher-order super-twisting design for a specific SE(3) spacecraft model, formulated through exponential-coordinate tracking errors
(Espindola and Tang, 2025)	General Lie groups	General intrinsic geometric sliding-mode framework for fully actuated mechanical systems, including the state-space Lie group and sliding-subgroup construction	Intrinsic Lie group configuration error and Lie-algebra-value velocity/sliding error	First-order SMC	General geometric framework, but based on a first-order reaching mechanism
(Moreno et al., 2022)	Euclidean vector systems	Robust finite-time multivariable super-twisting formulation with uncertain input matrix and perturbations	Euclidean vector-valued sliding variable	Multivariable STA	General multivariable super-twisting theory formulated in Euclidean vector spaces rather than on nonlinear configuration manifolds
(Moreno and Osorio, 2012)	Euclidean scalar systems	Strict Lyapunov construction for finite-time convergence, gain conditions, and reaching-time estimation	Scalar Euclidean sliding variable	Super-twisting	Methodological reference for the strict Lyapunov analysis underlying the present finite-time proof
Present work	General Lie groups	Intrinsic Lie-algebra-valued super-twisting formulation with covariant dynamics, strict metric Lyapunov analysis, constructive gain conditions, and finite-time reaching	Intrinsic Lie-group tracking error with Lie-algebra-valued sliding variable; no auxiliary configuration parametrization	Multivariable STA	Higher-order extension of a general intrinsic geometric sliding-mode framework, combining Lie-algebra/covariant reaching dynamics with a strict metric finite-time analysis

The remainder of the paper is organized as follows. Section 2 introduces the geometric notation, the state-space Lie-group structure, and the inherited sliding-subgroup construction. Section 3 presents the general mechanical model and the intrinsic multivariable Super-Twisting controller. Section 4 develops the nominal and matched-disturbance finite-time analyses and provides practical gain-selection conditions. Section 5 specializes the controller to spacecraft attitude dynamics on SO(3) and reports the comparative and Monte Carlo numerical results. Finally, Section 6 summarizes the main conclusions of the work.

## 2 Mathematical preliminaries

This section briefly reviews the geometric framework and notation adopted throughout the paper. The state-space Lie-group structure and the associated sliding-subgroup construction are adopted from (Espindola and Tang, 2025). These inherited results provide the geometric setting for the multivariable super-twisting reaching law developed in the subsequent sections.

### 2.1 Lie group geometry

Let G be a general finite-dimensional Lie group with identity element e∈G and associated Lie algebra

$$g≜T_{e}G,$$

identified with the tangent space at the identity. The tangent bundle of G is denoted by TG .

The configuration manifold is endowed with a left-invariant Riemannian metric

$$G_{g}:T_{g}G\times T_{g}G\to R,$$

whose restriction to the identity induces the inertia inner product

I:g×g→R.

The corresponding inertia norm is defined as

$$\|x{\|}_{I}≜\sqrt{I\left(x,x\right)},\;x\in g.$$ (1)

The associated flat and sharp operators are defined as

$$I^{♭}:g\to g^{*},\;I^{♯}:g^{*}\to g,$$

and satisfy

$$\left\langle I^{♭}\left(x\right),y\right\rangle =I\left(x,y\right),\;I^{♯}={\left(I^{♭}\right)}^{-1}.$$

### 2.2 Covariant differentiation

The left-invariant Levi–Civita connection associated with G restricts to the Lie algebra as

$$\nabla_{\xi }^{g}\zeta =\frac{1}{2}\left[\xi ,\zeta \right]-\frac{1}{2}I^{♯}\left({\text{ad }}_{\xi }^{*}I^{♭}\left(\zeta \right)+{\text{ad }}_{\zeta }^{*}I^{♭}\left(\xi \right)\right),\;\xi ,\zeta \in g.$$ (2)

For a Lie-algebra-valued function v(t)∈g evolving along a trajectory with left-trivialized velocity ξ(t)∈g , its covariant time derivative is

$$\nabla_{\xi }v=\dot{v}+\nabla_{\xi }^{g}v,$$ (3)

where $\dot{v}$ denotes the ordinary time derivative.

The Levi–Civita connection is metric compatible. Therefore, for any Lie-algebra-valued functions v(t),w(t)∈g evolving along the same trajectory,

$$\frac{d}{dt}I\left(v,w\right)=I\left(\nabla_{\xi }v,w\right)+I\left(v,\nabla_{\xi }w\right).$$ (4)

Property Equation 4 will be used explicitly in the Lyapunov analysis of the proposed super-twisting reaching law.

### 2.3 Tracking errors and state-space Lie group

Let $g_{d}\left(t\right)\in G$ be a desired trajectory satisfying

$${\dot{g}}_{d}=g_{d}\xi_{d},$$ (5)

where $\xi_{d}\left(t\right)\in g$ is its left-trivialized velocity. The left-invariant tracking errors are defined as

$$g_{e}=g_{d}^{-1}g,\;\eta_{d}={\text{Ad }}_{g_{e}^{-1}}\xi_{d},\;\nu_{e}=\xi -\eta_{d}.$$ (6)

Here, $g_{e}\in G$ denotes the intrinsic configuration error and $\nu_{e}\in g$ the corresponding left-trivialized velocity error. The complete tracking-error state is therefore given by

$$h_{e}≜\left(g_{e},\nu_{e}\right)\in TG≃G\times g.$$ (7)

The error kinematics then satisfy

$${\dot{g}}_{e}=g_{e}\nu_{e}.$$ (8)

We adopt the state-space Lie-group construction introduced in (Espindola and Tang, 2025). Let

$$\nu_{u}:G\to g$$

be a smooth kinematic feedback satisfying the assumptions stated therein, and let λ>0 be a design parameter. The tangent bundle TG≃G×g is endowed with the group operation

$$\left(g_{1},\nu_{1}\right)⋆\left(g_{2},\nu_{2}\right)=\left(g_{1}g_{2},\;\nu_{1}+\nu_{2}+\lambda \nu_{u}\left(g_{1}\right)+\lambda \nu_{u}\left(g_{2}\right)-\lambda \nu_{u}\left(g_{1}g_{2}\right)\right).$$ (9)

This inherited construction equips the tracking-error state space with a Lie-group structure.

### 2.4 Intrinsic set-valued sign map and Filippov solutions

The discontinuous terms appearing in the super-twisting reaching law are defined through the intrinsic set-valued sign map

$${\text{Sgn }}_{I}:g\to g,$$

given by

$${\text{Sgn }}_{I}\left(s\right)≜\left\{\begin{array}{cc} \frac{s}{\|s{\|}_{I}}, & s\neq 0,\\ \left\{z\in g:\|z{\|}_{I}\leq 1\right\}, & s=0. \end{array}\right.$$ (10)

For s≠0 , the map returns the normalized direction of s with respect to the inertia metric. At s=0 , it coincides with the closed unit ball in g induced by I .

Since the closed-loop dynamics contain the set-valued map Equation 10, they are interpreted as a differential inclusion. Throughout the paper, solutions are understood in the Filippov sense; namely, a solution is an absolutely continuous trajectory satisfying the corresponding differential inclusion for almost every time.

This formulation makes the discontinuous control term well defined at s=0 .

### 2.5 Sliding subgroup and reduced dynamics

Following (Espindola and Tang, 2025), the sliding variable is defined as the map

$$s:TG\to g,\;s\left(h_{e}\right)=\nu_{e}+\lambda \nu_{u}\left(g_{e}\right).$$ (11)

The associated sliding subgroup is defined as

$$H≜\left\{h_{e}\in TG:s\left(h_{e}\right)=0\right\}$$ (12)

The sliding variable $s\left(h_{e}\right)$ can be interpreted as the modified velocity-error coordinate induced by the state-space Lie-group construction. Accordingly, the covariant derivatives used to describe the reaching dynamics are taken along $s\left(h_{e}\right)$ . For a Lie-algebra-valued function v(t)∈g , we write

$$\nabla_{s}v≜\dot{v}+\nabla_{s\left(h_{e}\right)}^{g}v.$$ (13)

On the sliding subgroup H , the condition $s\left(h_{e}\right)=0$ implies

$$\nu_{e}=-\lambda \nu_{u}\left(g_{e}\right).$$ (14)

Substitution of Equation 14 into the error kinematics Equation 8 gives the reduced closed-loop dynamics

$${\dot{g}}_{e}=-\lambda g_{e}\nu_{u}\left(g_{e}\right).$$ (15)

Under the assumptions imposed on $\nu_{u}$ in (Espindola and Tang, 2025), the identity $g_{e}=e$ is an almost-globally asymptotically stable equilibrium of the reduced dynamics Equation 15. Hence, once the sliding subgroup is reached and remains invariant, the configuration error converges almost globally and asymptotically to $g_{e}=e$ , while $\nu_{e}$ converges to zero.

In the next section the contribution of this paper is addressed, which is the design of an intrinsic multivariable super-twisting reaching law that drives the system trajectories to the second-order sliding set in finite time.

## 3 Problem formulation and geometric super-twisting controller

This section develops the intrinsic multivariable Super-Twisting controller for fully actuated mechanical systems evolving on Lie groups. The system model and assumptions are first introduced, after which the desired covariant reaching dynamics are prescribed and the corresponding generalized control law is derived. The resulting finite-time reaching and tracking properties are then summarized before the detailed stability analysis of Section 4.

### 3.1 Problem formulation and system assumptions

Consider a fully actuated mechanical system evolving on a finite-dimensional Lie group G , with configuration g(t)∈G and left-trivialized velocity ξ(t)∈g . Its dynamics are given by

$$\dot{g}=g\xi ,$$ (16)

and

$$\dot{\xi }-I^{♯}\left({\text{ad }}_{\xi }^{*}I^{♭}\left(\xi \right)\right)=f_{u}+d\left(g,\xi ,t\right),$$ (17)

where $f_{u}\in g$ is the generalized control input and d(g,ξ,t)∈g is a matched generalized disturbance.

The system is assumed to be fully actuated, so that an arbitrary generalized input in g can be assigned through $f_{u}$ . The configuration g and the left-trivialized velocity ξ are assumed to be available for feedback.

The following assumptions are imposed throughout the controller design and stability analysis.

Assumption 1 *The desired trajectory* $g_{d}\left(t\right)$ *is sufficiently smooth. In particular,* $\xi_{d}$ *,* ${\dot{\xi }}_{d}$ *, and all reference-dependent quantities appearing in the feedback law are bounded.*

Assumption 2 *The map* $\nu_{u}:G\to g$ *is continuously differentiable and satisfies the assumptions of* (Espindola and Tang, 2025). *Consequently, the reduced kinematic dynamics on the sliding subgroup are almost globally asymptotically stable.*

Assumption 3 *The disturbance* d(g,ξ,t) *is matched, namely, it acts through the same channel as the control input* $f_{u}$ .

Two disturbance cases are considered. In the nominal case,

$$d\left(g,\xi ,t\right)=0.$$ (18)

In the perturbed case, d is locally absolutely continuous along the closed-loop trajectories and its covariant derivative satisfies

$${\left\|\nabla_{s}d\right\|}_{I}\leq \Delta_{d}$$ (19)

almost everywhere, for some known constant $\Delta_{d}\geq 0$ .

### 3.2 Intrinsic super-twisting controller design

The controller is constructed by first prescribing the desired intrinsic super-twisting reaching dynamics and then deriving the virtual-state dynamics and generalized control input required to realize them.

Introduce the auxiliary super-twisting state; v(t)∈g.

The desired intrinsic reaching dynamics are prescribed as

$$\nabla_{s}s=-k_{1}I{\left(s,s\right)}^{-1/4}s+v+d,\;\nabla_{s}v\in -k_{2}{\text{Sgn }}_{I}\left(s\right),$$ (20)

where $k_{1}>0$ and $k_{2}>0$ are the super-twisting gains.

For s≠0 , the first correction term can equivalently be written as

$$-k_{1}\frac{s}{\|s{\|}_{I}^{1/2}}.$$

This term is continuous and admits the value zero at s=0 , whereas the discontinuous correction acts only through the internal state v .

To obtain an implementable ordinary differential inclusion for the internal state, consider the covariant derivative of v directed along s ,

∇sv=v˙+∇sgv. (21)

The following result provides the ordinary differential inclusion used to implement the prescribed covariant dynamics.

Proposition 4 *Consider the virtual-state dynamics*

v˙∈−k2Sgn Is−∇sgv. (22)

Then every Filippov solution of Equation 22 satisfies the covariant differential inclusion Equation 20.

Proof. Substitution of Equation 22 into Equation 21 directly yields $\nabla_{s}v\in -k_{2}{\text{Sgn }}_{I}\left(s\right)$ .

Using the definition of the covariant derivative, the Levi–Civita connection in Equation 2, the identity [s,s]=0 , and the mechanical dynamics Equation 17, the sliding-variable dynamics can be written directly as

$$\nabla_{s}s=I^{♯}\left({\text{ad }}_{\xi }^{*}I^{♭}\left(\xi \right)\right)+f_{u}+d-{\dot{\eta }}_{d}+\lambda {\dot{\nu }}_{u}\left(g_{e}\right)-I^{♯}\left({\text{ad }}_{s}^{*}I^{♭}\left(s\right)\right).$$ (23)

Following the error-dynamics derivation adopted in (Espindola and Tang, 2025), the generalized control input is selected to cancel the known geometric, reference-dependent, and kinematic-feedback terms, while imposing the proposed super-twisting reaching law.

Proposition 5 *Consider the generalized control input*

$$f_{u}=-I^{♯}\left({\text{ad }}_{\xi }^{*}I^{♭}\left(\xi \right)\right)+I^{♯}\left({\text{ad }}_{s}^{*}I^{♭}\left(s\right)\right)+{\dot{\eta }}_{d}-\lambda {\dot{\nu }}_{u}\left(g_{e}\right)-k_{1}I{\left(s,s\right)}^{-1/4}s+v,$$ (24)

where the term $I{\left(s,s\right)}^{-1/4}s$ is defined as zero at s=0 by continuous extension. Then the resulting sliding dynamics satisfy

$$\nabla_{s}s=-k_{1}I{\left(s,s\right)}^{-1/4}s+v+d.$$ (25)

*Together with the virtual-state dynamics* Equation 22 *, the generalized input* Equation 24 *realizes the prescribed intrinsic super-twisting reaching law.*

Proof. Substitution of Equation 24 into Equation 23 cancels the known geometric, reference-dependent, and kinematic-feedback terms, directly yielding Equation 25.

### 3.3 Finite-time reaching and tracking properties

The purpose of the proposed controller is to drive the error trajectory to the inherited sliding subgroup

$$H=\left\{h_{e}\in TG:s\left(h_{e}\right)=0\right\}$$

through a second-order sliding motion. In the nominal case, this requires the finite-time convergence s→0 , v→0. In the presence of a matched disturbance, the corresponding second-order sliding condition is s→0, v+d→0, so that the internal super-twisting state compensates the disturbance according to. v→−d.

The main closed-loop property is stated below. Then, its finite-time proof and the corresponding gain conditions are developed in Section 4.

Theorem 6 *Consider the fully actuated mechanical system* Equations 16, 17 *under the generalized control law* Equation 24 *and the virtual-state dynamics* Equation 22 *. Suppose that* Assumptions 1–3 *hold and that* $k_{1}$ *and* $k_{2}$ *satisfy the sufficient gain conditions established in Section* 4*. Then the following properties hold.*

1. *In the nominal case* d=0 *, the variables* s *and* v *converge to zero in finite time. Consequently, the error state* $h_{e}$ *reaches the sliding subgroup* H *in finite time.*
2. *If the matched disturbance satisfies* Equation 19 *, the variables* s *and* v+d *converge to zero in finite time. Consequently,* s→0 *, and* v→−d.

3. *Once the corresponding second-order sliding condition is reached, it remains invariant in the Filippov sense.*
4. *Since* s=0 *implies* $h_{e}\in H$ *, the error state subsequently evolves according to the reduced kinematic dynamics recalled in Section* 2.5*. Therefore,* $\left(g_{e},\nu_{e}\right)\to \left(e,0\right)$ *almost globally and asymptotically.*

The finite-time result concerns the reaching of the second-order sliding condition and, consequently, of the sliding subgroup H . Convergence of the complete tracking error to (e,0) is instead almost global and asymptotic along the sliding motion.

## 4 Finite-time and robustness analysis

This section establishes the finite-time convergence properties of the proposed geometric super-twisting reaching law. First, finite-time convergence is proved in the nominal case. Afterwards, exact finite-time compensation of a regular matched disturbance is established. All differential inequalities are understood almost everywhere along Filippov solutions.

Continuity of the Lyapunov function alone is not sufficient to justify their integration across zero. Therefore, following the strategy used for the classical scalar super-twisting algorithm in (Moreno and Osorio, 2012), absolute continuity of the Lyapunov function along the closed-loop trajectories is established below.

### 4.1 Nominal finite-time analysis

Consider the nominal closed-loop reaching dynamics

$$\nabla_{s}s=-k_{1}I{\left(s,s\right)}^{-1/4}s+v,\;\nabla_{s}v\in -k_{2}{\text{Sgn }}_{I}\left(s\right).$$ (26)

For s≠0 , the second equation reduces to

$$\nabla_{s}v=-k_{2}\frac{s}{I{\left(s,s\right)}^{1/2}}.$$

For clarity, introduce

$$\mu_{1}≜I{\left(s,s\right)}^{1/4},\;\mu_{2}≜I{\left(v,v\right)}^{1/2},\;\mu ≜\left[\begin{array}{c} \mu_{1}\\ \mu_{2} \end{array}\right].$$ (27)

Thus,

$$I{\left(s,s\right)}^{1/2}=\mu_{1}^{2},\;I\left(v,v\right)=\mu_{2}^{2}.$$

Define the Lyapunov candidate

$$V=\alpha_{1}I{\left(s,s\right)}^{1/2}+2\alpha_{12}I{\left(s,s\right)}^{-1/4}I\left(s,v\right)+\alpha_{2}I\left(v,v\right),$$ (28)

where $\alpha_{1}>0$ , $\alpha_{2}>0,$ and $\alpha_{12}<0$ are auxiliary Lyapunov-construction parameters and do not enter the implemented control law. The actual controller gains are $k_{1}$ and $k_{2}$ , introduced in Section 3.2. The role of the parameters $\alpha_{i}$ is solely to construct a Lyapunov function satisfying the positive-definiteness conditions required by the convergence analysis.

By the Cauchy–Schwarz inequality induced by I ,

$$\left|I\left(s,v\right)\right|\leq I{\left(s,s\right)}^{1/2}I{\left(v,v\right)}^{1/2}=\mu_{1}^{2}\mu_{2}.$$

Consequently,

$$I{\left(s,s\right)}^{-1/4}\left|I\left(s,v\right)\right|\leq \mu_{1}\mu_{2}.$$

Since $\alpha_{12}<0$ , the Lyapunov function satisfies

$$\mu^{T}P\mu \leq V\leq \mu^{T}\tilde{P}\mu ,$$ (29)

where

$$P=\left[\begin{array}{cc} \alpha_{1} & \alpha_{12}\\ \alpha_{12} & \alpha_{2} \end{array}\right],\;\tilde{P}=\left[\begin{array}{cc} \alpha_{1} & -\alpha_{12}\\ -\alpha_{12} & \alpha_{2} \end{array}\right].$$ (30)

Both matrices are positive definite if $\alpha_{1}\alpha_{2}>\alpha_{12}^{2}$ .

Moreover,

$$\tilde{P}=DPD,\;D=\text{diag }\left(1,-1\right),$$

and therefore P and $\tilde{P}$ have the same eigenvalues. It follows that

$$\lambda_{\min}\left(P\right)\|\mu {\|}^{2}\leq V\leq \lambda_{\max}\left(P\right)\|\mu {\|}^{2}.$$ (31)

Although the cross term in Equation 28 is formally undefined at s=0 , the preceding bound shows that it converges to zero as s→0 , provided that v remains bounded. Hence, V admits a continuous extension at s=0 .

The following lemma supplies the additional regularity needed to integrate the Lyapunov inequality. It is stated in a form that applies both to the nominal case, with y=v , and to the matched-disturbance case, with y=w=v+d .

Lemma 7 (Absolute continuity along Filippov solutions). *Let* $y\in AC_{\text{loc}}\left(\left[0,\infty \right);g\right)$ *and consider the first-layer dynamics*

$$\nabla_{s}s=-k_{1}I{\left(s,s\right)}^{-1/4}s+y,$$ (32)

where $I{\left(s,s\right)}^{-1/4}s$ is defined as zero at s=0 . Define

$$\Phi \left(s\right)≜\left\{\begin{array}{cc} I{\left(s,s\right)}^{-1/4}s, & s\neq 0,\\ 0, & s=0, \end{array}\right.$$ (33)

and

$$V_{y}\left(s,y\right)≜\alpha_{1}I{\left(s,s\right)}^{1/2}+2\alpha_{12}I\left(\Phi \left(s\right),y\right)+\alpha_{2}I\left(y,y\right).$$ (34)

Let

$$T_{⋆}≜\inf\left\{t\geq 0:\left(s\left(t\right),y\left(t\right)\right)=\left(0,0\right)\right\},\;\inf\emptyset ≜+\infty .$$ (35)

*Then* $t\mapsto V_{y}\left(s\left(t\right),y\left(t\right)\right)$ *is absolutely continuous on every compact interval* $\left[0,T\right]\subset \left[0,T_{⋆}\right)$ .

Proof. Since s and y are absolutely continuous, the first and third terms in Equation 34 are absolutely continuous on compact intervals. Thus, it remains to consider

$$C\left(t\right)=I\left(\Phi \left(s\left(t\right)\right),y\left(t\right)\right).$$

Fix $T<T_{⋆}$ and let $t_{0}\in \left[0,T\right]$ satisfy $s\left(t_{0}\right)=0$ . By the definition of $T_{⋆}$ , $y\left(t_{0}\right)\neq 0$ . Set

$$e_{0}=\frac{y\left(t_{0}\right)}{\|y\left(t_{0}\right){\|}_{I}}.$$

Using Equation 13, the ordinary derivative of s satisfies, almost everywhere,

s˙=−k1Φs+y−∇sgs. (36)

The maps Φ(s) and ∇sgs vanish as s→0 , while y is continuous. Hence, after possibly reducing a neighborhood of $t_{0}$ , there exists c>0 such that

$$I\left(e_{0},\dot{s}\left(t\right)\right)\geq c$$

for almost every t in that neighborhood. Integration on either side of $t_{0}$ gives

$$\|s\left(t\right){\|}_{I}\geq \left|I\left(e_{0},s\left(t\right)\right)\right|\geq c|t-t_{0}|.$$ (37)

Moreover, the right-hand side of Equation 36 is locally bounded. Therefore, for some M>0 ,

$$\left\|s\left(t\right){\|}_{I}\leq M|t-t_{0}\right|$$ (38)

near $t_{0}$ .

For s(t)≠0 , differentiation of Equation 33 yields

$$\dot{\Phi }\left(s\left(t\right)\right)=I{\left(s,s\right)}^{-1/4}\dot{s}-\frac{1}{2}I{\left(s,s\right)}^{-5/4}I\left(s,\dot{s}\right)s.$$ (39)

Consequently,

$$\|\dot{\Phi }\left(s\left(t\right)\right){\|}_{I}\leq \frac{3}{2}I{\left(s\left(t\right),s\left(t\right)\right)}^{-1/4}\|\dot{s}\left(t\right){\|}_{I}\leq \frac{K}{|t-t_{0}{|}^{1/2}}$$ (40)

for some K>0 . Since $|t-t_{0}{|}^{-1/2}$ is locally integrable and $\Phi \left(s\left(t\right)\right)\to 0=\Phi \left(s\left(t_{0}\right)\right)$ , the map t↦Φ(s(t)) is absolutely continuous through $t_{0}$ .

Every zero of s in [0,T] is isolated by Equation 37. Since the zero set is closed, compactness then implies that s has only finitely many zeros in [0,T] . It follows that Φ(s(⋅)) is absolutely continuous on the whole interval. Finally, y is absolutely continuous and both factors are bounded on [0,T] , so C(t) is absolutely continuous. This proves the claim.

Remark 8 The role of Lemma 7 is analogous to the absolute-continuity argument in (Moreno and Osorio, 2012) and similar to the one used in (Seeber et al., 2018). In the scalar super-twisting algorithm, monotonicity of the first state while it crosses zero is used to handle the non-Lipschitz transformed variable. In the present multivariable setting there is no scalar ordering; monotonicity is replaced by the transversal estimate Equation 37, obtained by projecting the dynamics onto the direction $y\left(t_{0}\right)$ .

Using metric compatibility and the nominal dynamics Equation 26, direct differentiation of Equation 28 gives

$$\dot{V}=\frac{1}{\mu_{1}}\left[-c_{1}\mu_{1}^{2}+c_{12}\frac{I\left(s,v\right)}{\mu_{1}}-\frac{\alpha_{12}I{\left(s,v\right)}^{2}}{\mu_{1}^{4}}+2\alpha_{12}\mu_{2}^{2}\right],$$ (41)

where $c_{1}=\alpha_{1}k_{1}+2\alpha_{12}k_{2}$ , and $c_{12}=\alpha_{1}-\alpha_{12}k_{1}-2\alpha_{2}k_{2}$ .

Since $\alpha_{12}<0$ , application of the Cauchy–Schwarz inequality to the mixed terms in Equation 41 yields the quadratic upper bound

$$\dot{V}\leq -\frac{1}{\mu_{1}}\mu^{T}Q\mu ,$$ (42)

with

$$Q=\left[\begin{array}{cc} c_{1} & -\frac{\left|c_{12}\right|}{2}\\ -\frac{\left|c_{12}\right|}{2} & -\alpha_{12} \end{array}\right].$$ (43)

Because $-\alpha_{12}>0$ , Sylvester’s criterion shows that Q≻0 if and only if

$$c_{1}>0,\;c_{1}\left(-\alpha_{12}\right)>\frac{c_{12}^{2}}{4}.$$ (44)

Theorem 9 (Nominal finite-time reaching). *Suppose that* P≻0 *and* Q≻0 *. Then every Filippov solution of* Equation 26 *satisfies* (s(t),v(t))→(0,0) *in finite time. An upper bound on the reaching time is*

$$T_{0}\leq \frac{2\lambda_{\max}{\left(P\right)}^{1/2}V{\left(0\right)}^{1/2}}{\lambda_{\min}\left(Q\right)}.$$ (45)

Proof.

From Equation 42, the Rayleigh bound and $\mu_{1}\leq \|\mu \|$ give

$$\dot{V}\leq -\lambda_{\min}\left(Q\right)\|\mu \|.$$

Using the upper Lyapunov bound in Equation 31, $\|\mu \|\geq {\left(V/\lambda_{\max}\left(P\right)\right)}^{1/2}$ , and therefore

$$\dot{V}\leq -\frac{\lambda_{\min}\left(Q\right)}{\lambda_{\max}{\left(P\right)}^{1/2}}V^{1/2}$$ (46)

almost everywhere before the first hitting time of (s,v)=(0,0) .

Since V is absolutely continuous by Lemma 7, integration of Equation 46 gives

$$V{\left(t\right)}^{1/2}\leq \max\left\{0,\;V{\left(0\right)}^{1/2}-\frac{\lambda_{\min}\left(Q\right)}{2\lambda_{\max}{\left(P\right)}^{1/2}}t\right\}.$$

Hence the first hitting time is finite and satisfies Equation 45.

The nominal feasibility conditions admit a particularly simple gain-first construction. Let the implemented controller gains $k_{1}>0$ and $k_{2}>0$ be given. A convenient set of auxiliary Lyapunov parameters is

$$\alpha_{12}=-1,\;\alpha_{1}=\frac{4k_{2}}{k_{1}},\;\alpha_{2}=\frac{2}{k_{1}}+\frac{k_{1}}{2k_{2}}.$$ (47)

These quantities are constructed only after the controller gains have been selected and serve exclusively as a stability certificate. Indeed,

$$\alpha_{1}\alpha_{2}-\alpha_{12}^{2}=1+\frac{8k_{2}}{k_{1}^{2}}>0,$$ (48)

and therefore P≻0 . Moreover, $c_{12}=0$ and $c_{1}=2k_{2}$ , thus yielding

$$Q=\left[\begin{array}{cc} 2k_{2} & 0\\ 0 & 1 \end{array}\right]≻0.$$ (49)

Thus, every suitable positive controller-gain pair $\left(k_{1},k_{2}\right)$ admits this particular nominal Lyapunov certificate.

### 4.2 Matched-disturbance rejection

Consider now the closed-loop dynamics in the presence of the matched disturbance d :

$$\nabla_{s}s=-k_{1}I{\left(s,s\right)}^{-1/4}s+v+d,\;\nabla_{s}v\in -k_{2}{\text{Sgn }}_{I}\left(s\right).$$ (50)

Define, for analysis purposes only, w≜v+d . The variable w is not required for implementation and does not require measurement of the disturbance. It is introduced only to characterize the disturbance-compensation property of the internal state v .

Using the linearity of the covariant derivative, Equation 50 becomes

$$\nabla_{s}s=-k_{1}I{\left(s,s\right)}^{-1/4}s+w,\;\nabla_{s}w\in -k_{2}{\text{Sgn }}_{I}\left(s\right)+\nabla_{s}d.$$ (51)

Under the disturbance regularity condition Equation 19, consider the same Lyapunov construction used in the nominal case, with v replaced by w :

$$V_{d}=\alpha_{1}I{\left(s,s\right)}^{1/2}+2\alpha_{12}I{\left(s,s\right)}^{-1/4}I\left(s,w\right)+\alpha_{2}I\left(w,w\right),$$ (52)

with

$$\mu =\left[\begin{array}{c} I{\left(s,s\right)}^{1/4}\\ I{\left(w,w\right)}^{1/2} \end{array}\right].$$

The same quadratic bounds as in Equation 31 therefore hold:

$$\lambda_{\min}\left(P\right)\|\mu {\|}^{2}\leq V_{d}\leq \lambda_{\max}\left(P\right)\|\mu {\|}^{2}.$$ (53)

Since w=v+d is locally absolutely continuous, Lemma 7 applies directly with y=w . Using (19) and the Cauchy–Schwarz inequality to bound the additional terms generated by $\nabla_{s}d$ yields

$${\dot{V}}_{d}\leq -\frac{1}{\mu_{1}}\mu^{T}\left(Q-R_{d}\right)\mu ,$$ (54)

where

$$R_{d}=\left[\begin{array}{cc} 2|\alpha_{12}|\Delta_{d} & \alpha_{2}\Delta_{d}\\ \alpha_{2}\Delta_{d} & 0 \end{array}\right].$$ (55)

Theorem 10 (Exact matched-disturbance rejection). *Suppose that* P≻0 *, the disturbance satisfies* Equation 19 *, and the controller gains are selected such that* $Q-R_{d}≻0.$ *Then* (s(t),w(t))→(0,0) *in finite time. An upper bound on the reaching time is*

$$T_{d}\leq \frac{2\lambda_{\max}{\left(P\right)}^{1/2}V_{d}{\left(0\right)}^{1/2}}{\lambda_{\min}\left(Q-R_{d}\right)}.$$ (56)

Consequently,

$$s\left(t\right)\to 0,\;v\left(t\right)\to -d\left(t\right)$$ (57)

in finite time.

Proof.

From Equation 54, the Rayleigh bound, $\mu_{1}\leq \|\mu \|$ , and Equation 53 give

$${\dot{V}}_{d}\leq -\frac{\lambda_{\min}\left(Q-R_{d}\right)}{\lambda_{\max}{\left(P\right)}^{1/2}}V_{d}^{1/2}$$ (58)

almost everywhere before the first hitting time of (s,w)=(0,0) .

Absolute continuity of $V_{d}$ and integration of Equation 58 then give the finite-time bound Equation 56. Since w=v+d , convergence of (s,w) to (0, 0) directly implies Equation 57.

For a prescribed disturbance-rate bound $\Delta_{d}$ , the gain-first construction Equation 47 also provides a direct robust feasibility test. With these auxiliary Lyapunov parameters, Equation 49 and Equation 55 give

$$Q-R_{d}=\left[\begin{array}{cc} 2\left(k_{2}-\Delta_{d}\right) & -\alpha_{2}\Delta_{d}\\ -\alpha_{2}\Delta_{d} & 1 \end{array}\right],\;\text{where}\;\alpha_{2}=\frac{2}{k_{1}}+\frac{k_{1}}{2k_{2}}.$$ (59)

For this particular certificate, the robust condition $Q-R_{d}≻0$ is therefore equivalent to

$$2\left(k_{2}-\Delta_{d}\right)>{\left(\frac{2}{k_{1}}+\frac{k_{1}}{2k_{2}}\right)}^{2}\Delta_{d}^{2}.$$ (60)

From a practical tuning perspective, the theoretical inequalities should therefore be used as feasibility constraints. A useful tuning workflow is the following. First, a candidate pair $\left(k_{1},k_{2}\right)$ is selected and the corresponding Lyapunov parameters are generated through Equation 47. If a certified disturbance-rate bound is available, the pair is retained only if Equation 60 is satisfied with an adequate positive margin.

Among feasible gain pairs, $k_{1}$ acts primarily as a transient-performance parameter. The gain-sensitivity results reported in Section 5.3.5 show that increasing $k_{1}$ substantially reduces the reaching time over the investigated range, but excessive values increase control effort, peak torque, and residual angular-rate activity. The gain $k_{2}$ has a weaker influence on reaching time over the tested range, while increasing it generally increases control effort and angular-rate RMSE. At the same time, for fixed $k_{1}$ , increasing $k_{2}$ relaxes the robust feasibility condition Equation 60. A practical choice is therefore to select $k_{2}$ sufficiently large to provide the required robust feasibility margin, and subsequently adjust $k_{1}$ to meet the desired reaching-time requirement while monitoring actuator effort, peak torque, and steady-state tracking performance.

Corollary 11 (Second-order sliding and tracking). *Under the assumptions of either Theorem* 9 *or Theorem* 10*, the closed-loop trajectory reaches* s=0,  $\nabla_{s}s=0$ *in finite time.*

Since $s=0\;⟺\;h_{e}\in H,$ the tracking-error state reaches the sliding subgroup H in finite time. Thereafter, the reduced dynamics on H imply $\left(g_{e},\nu_{e}\right)\to \left(e,0\right)$ almost globally and asymptotically.

Proof.In the nominal case, finite-time convergence gives s=0 and v=0 . In the matched-disturbance case, it gives s=0 and v+d=0 . In both cases, substitution into the first-layer dynamics yields $\nabla_{s}s=0.$ The tracking conclusion then follows from the inherited stability properties of the reduced dynamics on H . □

## 5 Numerical simulations

This section provides a numerical validation of the proposed control framework for spacecraft attitude tracking on SO(3) . First, the general control law is specialized to rigid-spacecraft attitude dynamics. Its performance is then compared with classical first-order sliding-mode controllers under identical operating conditions. Finally, Monte Carlo robustness and gain-sensitivity studies are presented to assess convergence, tracking accuracy, control effort, and sensitivity to modeling and actuator uncertainties.

### 5.1 Reaching law for spacecraft attitude control on SO(3)

For the numerical validation, the proposed controller is specialized to the attitude dynamics of a rigid spacecraft. Let R(t)∈SO(3) denote the spacecraft attitude and let $\Omega \left(t\right)\in R^{3}$ be its body angular velocity. The equations of motion are

$$\dot{R}=R\hat{\Omega },$$ (61)

and

$$J\dot{\Omega }+\Omega \times J\Omega =\tau +d_{\tau },$$ (62)

where $J=J^{T}≻0$ is the inertia matrix, $\tau \in R^{3}$ is the commanded control torque, and $d_{\tau }\in R^{3}$ is a matched disturbance torque.

The inertia-induced inner product on $R^{3}≃so\left(3\right)$ is

$$I\left(x,y\right)=x^{T}Jy.$$ (63)

Let $R_{d}\left(t\right)\in SO\left(3\right)$ be the desired attitude, with body angular velocity $\Omega_{d}\left(t\right)$ defined by

$${\dot{R}}_{d}=R_{d}{\hat{\Omega }}_{d}.$$ (64)

Define the attitude and angular-velocity errors as

$$R_{e}=R_{d}^{T}R,\;\Omega_{e}=\Omega -\sigma ,\;\sigma =R_{e}^{T}\Omega_{d}.$$ (65)

The corresponding error kinematics are

$${\dot{R}}_{e}=R_{e}{\hat{\Omega }}_{e},$$ (66)

while

$$\dot{\sigma }=R_{e}^{T}{\dot{\Omega }}_{d}-\Omega_{e}\times \sigma .$$ (67)

The kinematic feedback is selected through the logarithmic map on SO(3) as

$$\Omega_{u}\left(R_{e}\right)=\log{\left(R_{e}\right)}^{\vee }.$$ (68)

For $\text{tr }\left(R_{e}\right)\neq -1$ , it admits the closed-form expression

$$\Omega_{u}\left(R_{e}\right)=\frac{\theta_{e}}{2\sin\theta_{e}}{\left(R_{e}-R_{e}^{T}\right)}^{\vee },\;\theta_{e}=\cos^{-1}\left(\frac{\text{tr }\left(R_{e}\right)-1}{2}\right).$$ (69)

For the SO(3) specialization, the kinematic feedback is smooth on the open dense domain SO(3)\C , where

$$C=\left\{R:\text{tr }R=-1\right\}.$$

This exclusion is consistent with the almost-global nature of the tracking result.

Since the error kinematics satisfy ${\dot{R}}_{e}=R_{e}{\hat{\Omega }}_{e}$ , the differential of the logarithmic map gives

$${\dot{\Omega }}_{u}\left(R_{e}\right)=J_{r}^{-1}\left(\Omega_{u}\left(R_{e}\right)\right)\Omega_{e},$$ (70)

where $J_{r}^{-1}$ is the inverse right Jacobian of the exponential map on SO(3) (Chirikjian, 2012). Its closed-form expression is

$$J_{r}^{-1}\left(\phi \right)=I+\frac{1}{2}\hat{\phi }+\left(\frac{1}{\theta^{2}}-\frac{1+\cos\theta }{2\theta \sin\theta }\right){\hat{\phi }}^{\;2},\;\phi =\Omega_{u}\left(R_{e}\right),\;\theta =\|\phi \|.$$ (71)

At ϕ=0 , the Jacobian is defined by continuous extension as $J_{r}^{-1}\left(0\right)=I$ . The expression is exact on the regular domain of the logarithmic map.

The attitude sliding variable is therefore

$$s=\Omega_{e}+\lambda \Omega_{u}\left(R_{e}\right).$$ (72)

The detailed specialization of the intrinsic controller to SO(3) , including the inertia-induced Levi–Civita connection, the explicit virtual-state dynamics, and the algebra leading from the generalized Lie-group control law to the commanded spacecraft torque, is reported in Supplementary Appendix.

The virtual-state dynamics are given by

$$\dot{v}\in -k_{2}{\text{Sgn }}_{J}\left(s\right)-\nabla_{s}^{so\left(3\right)}v,$$ (73)

where the explicit expression of the connection and of the metric sign map are given in Supplementary Appendix.

Specialization of the generalized control law Equation 24 yields the commanded body torque

$$\begin{array}{ll} \tau = & \;-\left(J\Omega \right)\times \Omega +\left(Js\right)\times s\\ & \;+J\left[\dot{\sigma }-\lambda {\dot{\Omega }}_{u}\left(R_{e}\right)-k_{1}{\left(s^{T}Js\right)}^{-1/4}s+v\right]. \end{array}$$ (74)

The homogeneous term ${\left(s^{T}Js\right)}^{-1/4}s$ is assigned the value zero at s=0 by continuous extension.

As shown in Supplementary Appendix, substitution of Equation 74 into the spacecraft dynamics gives

$$\nabla_{s}s=-k_{1}{\left(s^{T}Js\right)}^{-1/4}s+v+J^{-1}d_{\tau },$$ (75)

together with Equation 73. Hence, the general finite-time and robustness results apply with the generalized disturbance

$$d=J^{-1}d_{\tau }.$$ (76)

### 5.2 Comparison with classical sliding-mode control

#### 5.2.1 Simulation setup

The proposed Super-Twisting Geometric Sliding-Mode Controller (*ST-GSMC*) is compared with two first-order geometric sliding-mode controllers derived from the GSMC formulation introduced in (Espindola and Tang, 2025). All three controllers employ the same attitude sliding variable

$$s=\Omega_{e}+\lambda \Omega_{u}\left(R_{e}\right),$$

so that the comparison focuses on the different reaching mechanisms rather than on different sliding-manifold definitions.

The first benchmark, denoted by *GSMC*, corresponds to the discontinuous fixed-time reaching law obtained by selecting the exponent q=1/2 in the first-order GSMC formulation. In this case, the singular power term reduces, for s≠0 , to the metric sign map ${\text{Sgn }}_{J}\left(s\right)$ and is interpreted at s=0 in the Filippov sense.

The second benchmark, denoted by *GSMC-cont*, uses instead an exponent $0<q_{c}<1/2$ . The resulting reaching term converges continuously to zero as s→0 , thereby reducing the discontinuous switching action at the expense of replacing exact convergence to the sliding manifold with convergence to a neighborhood under the corresponding formulation.

For completeness, the explicit commanded-torque expressions of both benchmark controllers, together with the general first-order GSMC law from which they are obtained, are reported in Supplementary Appendix. This keeps the present section focused on the simulation conditions and the quantitative controller comparison.

All controllers are evaluated under the same initial conditions, spacecraft inertia, reference motion, actuator configuration, and disturbance model. The considered task is an attitude-tracking maneuver on SO(3) . The reference attitude is propagated according to a constant body-fixed reference angular velocity $\omega_{d}$ , while the spacecraft starts from a large initial attitude error and zero angular velocity. A bounded sinusoidal external disturbance torque is applied throughout the simulation to evaluate the robustness of the controllers under identical perturbations. It is defined as

$$d_{\tau }\left(t\right)=2.5\times 10^{-3}\left[\begin{array}{c} 0.8190\\ 0.0875\\ 0.5670 \end{array}\right]\sin\left(t+\frac{\pi }{2}\right)\;N\;m.$$ (77)

The kinematic gain λ is common to the three controllers. The super-twisting gains are selected as

$$k_{1}=0.075,\;k_{2}=0.00625.$$

Their Lyapunov feasibility is verified directly using the matrix conditions derived in Section 4. In the nominal case, the choice

$$\alpha_{1}=0.2,\;\alpha_{2}=22,\;\alpha_{12}=-1$$

gives

$$\alpha_{1}\alpha_{2}-\alpha_{12}^{2}=3.4>0,\;\lambda_{\min}\left(P\right)=0.1542,\;\lambda_{\min}\left(Q\right)=2.5\times 10^{-3},$$

and therefore satisfies P≻0 and Q≻0 .

For the sinusoidal disturbance considered in the comparison an admissible robust certificate is obtained with

$$\alpha_{1}=0.9189,\;\alpha_{2}=79.5111,\;\alpha_{12}=-1,$$

for which

$$\lambda_{\min}\left(P\right)=0.9062,\;\lambda_{\min}\left(Q-R_{d}\right)=1.3769\times 10^{-2}>0.$$

Hence, the matrix feasibility conditions are satisfied with a strict positive margin for the adopted disturbance-rate value.

The control torque is generated by a redundant four-reaction-wheel assembly arranged in a symmetric pyramidal configuration. The wheel-axis matrix is defined using an elevation angle $\beta =\tan^{-1}\left(1/\sqrt{2}\right)≃35.264{}^{\circ}$ and azimuth angles $\psi_{i}=\left\{0{}^{\circ},90{}^{\circ},180{}^{\circ},270{}^{\circ}\right\}$ . Each wheel is subject to the torque and angular-momentum constraints

$$|\tau_{w,i}|\leq 0.025\;N\;m,\;|h_{w,i}|\leq 0.6\;N\;m\;s,\;i=1,\ldots ,4.$$ (78)

An actuation delay of 0.01 s, corresponding to one simulation time step, is also included in the reaction-wheel model. The simulation and controller parameters are summarized in Table 2. The controllers were tuned to complete the maneuver without actuator saturation.

**TABLE 2:** Simulation and controller parameters.

Parameter	Value
Simulation timing	
Integration step dt	0.01 s
Simulation horizon T	1500 s
Spacecraft model	
Inertia matrix J	diag (1,0.5,1.25) kg $\cdot {\text{m}}^{2}$
Initial attitude R(0)	$R_{\text{eul}}\left(-\pi /2,\;\pi /3,\;\pi /5\right)$
Initial angular velocity ω(0)	${\left(0,\;0,\;0\right)}^{⊤}$ rad/s
Reference trajectory	
Initial reference attitude $R_{d}\left(0\right)$	$I_{3}$
Reference angular velocity $\omega_{d}$	${\left(0,\;0,\;0.1\right)}^{⊤}$ rad/s
External disturbance	
Disturbance torque d(t)	$2.5\times 10^{-3}{\left[\begin{array}{ccc} 0.8190 & 0.0875 & 0.5670 \end{array}\right]}^{\;⊤}\sin\;\left(t+\frac{\pi }{2}\right)$ N ⋅ m
GSMC	
Sliding-surface parameter λ	0.01
Reaching-law gain $k_{s}$	0.01
Reaching-law exponent σ	0.5
GSMC-cont	
Sliding-surface parameter λ	0.01
Reaching-law gain $k_{s}$	0.08
Reaching-law exponent σ	0.25
ST-GSMC	
Sliding-surface parameter λ	0.01
Super-twisting gain $k_{1}$	0.075
Super-twisting gain $k_{2}$	0.00625

The spacecraft attitude is propagated using a fourth-order Lie-group Runge–Kutta method on SO(3) . At each integration step, the incremental rotations are composed through the matrix exponential, thereby preserving the orthogonality of the rotation matrix. The angular-velocity dynamics are integrated through the corresponding fourth-order Runge–Kutta update.

#### 5.2.2 Comparison results

Figure 1 presents the attitude-tracking error in terms of the equivalent rotation angle associated with $R_{d}^{⊤}R$ . All three controllers reduce the large initial attitude error and drive the spacecraft toward the desired trajectory. The discontinuous GSMC and the proposed ST-GSMC attain an error close to zero, whereas the continuous GSMC implementation converges to a small residual neighborhood. This behavior is consistent with the replacement of the discontinuous reaching term by a continuous approximation.

**FIGURE 1:**
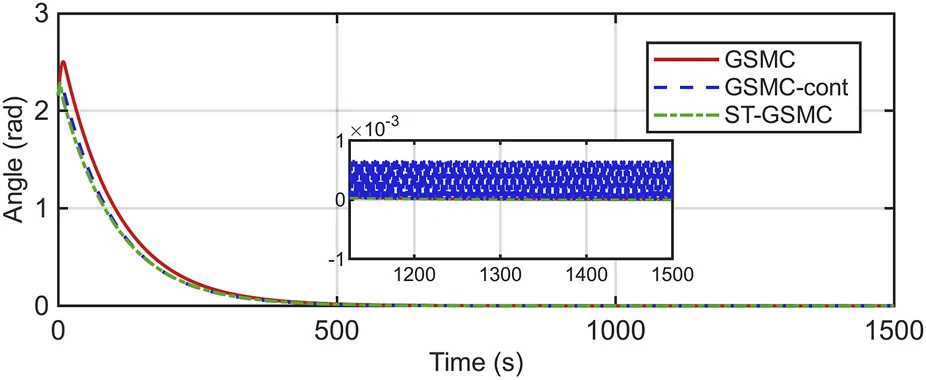
Equivalent rotation-angle tracking error obtained with the GSMC, GSMC-cont, and ST-GSMC controllers. The inset magnifies the steady-state response and highlights the residual tracking-error oscillations.

The corresponding angular-velocity responses are shown in Figure 2. The three controllers track the commanded reference angular velocity after the initial transient. The main differences between the controllers are therefore visible in the transient response and in the magnitude of the residual steady-state oscillations. The actuator effort is compared in Figure 3, which reports the torques allocated to the four reaction wheels. The discontinuous GSMC produces the most evident high-frequency switching activity. The continuous GSMC substantially reduces this behavior, while the proposed ST-GSMC provides a similarly smooth actuator command without the steady-state accuracy degradation associated with the continuous first-order approximation. Only the initial portion of the simulation is displayed in this figure, since it contains the relevant reaching transient and makes the differences in actuator activity easier to distinguish. Figure 4 shows the evolution of the three components of the sliding variable. All controllers drive the system toward the prescribed sliding manifold. The discontinuous GSMC reaches the manifold rapidly but retains a visible high-frequency oscillation around it. The continuous GSMC reduces the switching activity at the price of a larger residual neighborhood. The proposed ST-GSMC reaches a narrow neighborhood of the manifold with limited overshoot and reduced high-frequency activity. The insets magnify the reaching phase and make the differences between the three control laws more visible than in the full simulation interval. Further insight into the reaching dynamics is provided by Figure 5, which reports the numerical time derivative of the sliding variable. The discontinuous GSMC produces a persistent high-frequency derivative, as expected from its switching control action. The continuous GSMC reduces this effect, although residual oscillations remain. In contrast, after the initial transient, the ST-GSMC derivative remains confined to a comparatively small neighborhood of zero.

**FIGURE 2:**
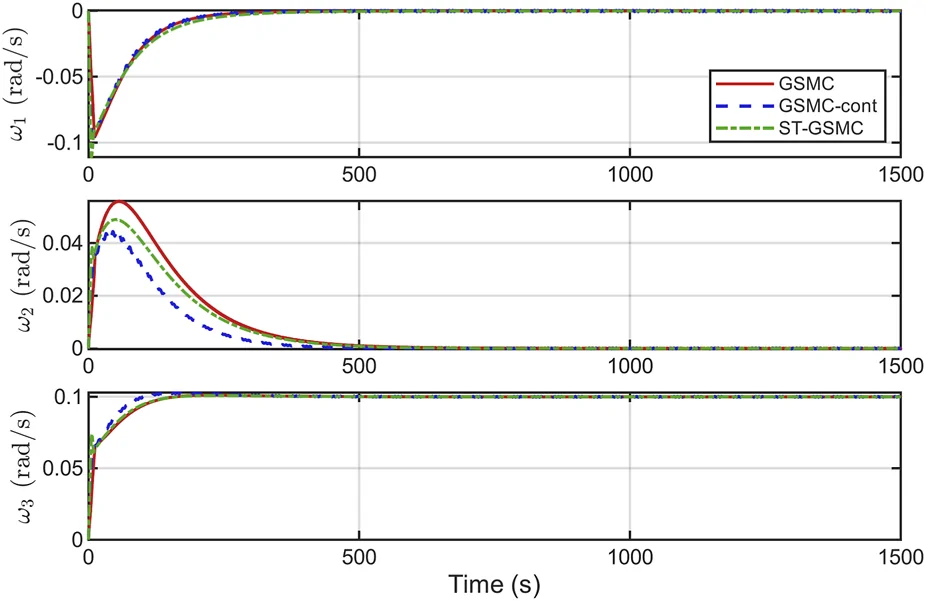
Body angular-velocity components obtained with the three controllers. The panels show $\omega_{1}$ , $\omega_{2}$ , and $\omega_{3}$ , respectively. All controllers track the prescribed reference angular velocity, with different transient and steady-state characteristics.

**FIGURE 3:**
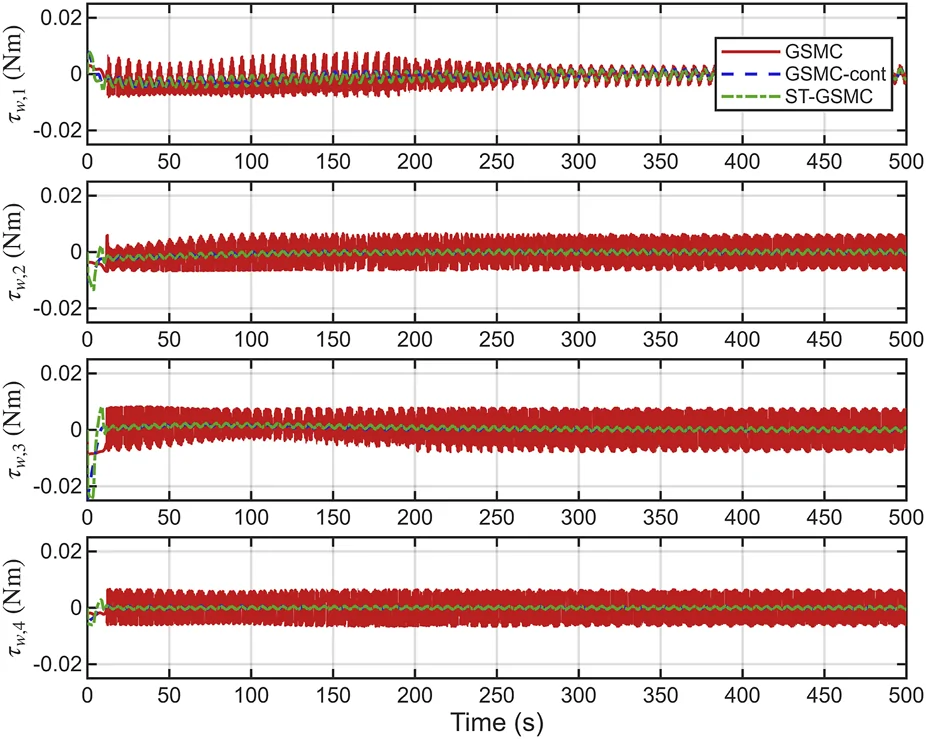
Reaction-wheel torque histories generated by the three controllers during the initial maneuver transient. Each panel corresponds to one wheel of the four-wheel pyramidal configuration. The discontinuous GSMC exhibits the largest high-frequency switching activity.

**FIGURE 4:**
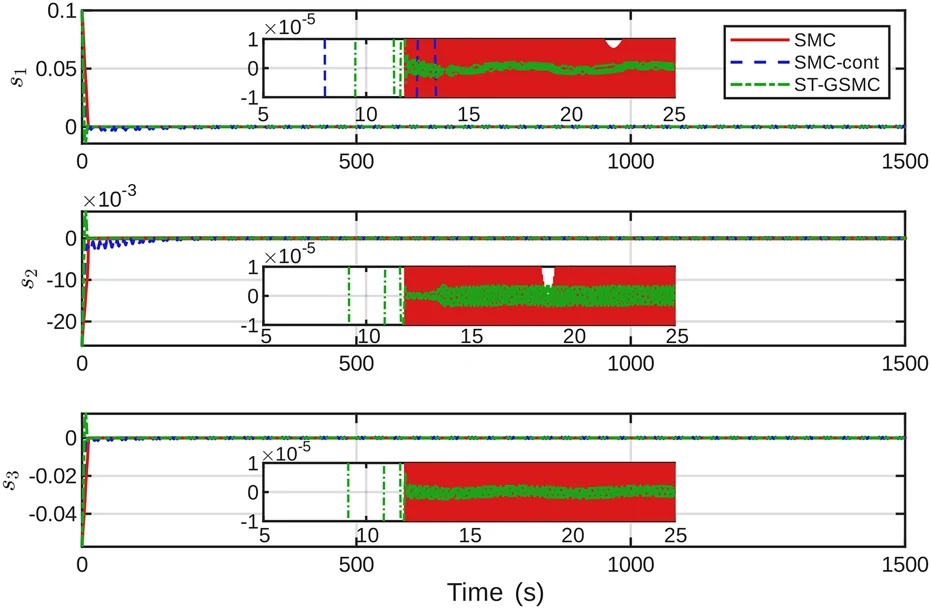
Sliding-variable components for the GSMC, GSMC-cont, and ST-GSMC controllers. The insets magnify the reaching transient and the residual motion around the sliding manifold.

**FIGURE 5:**
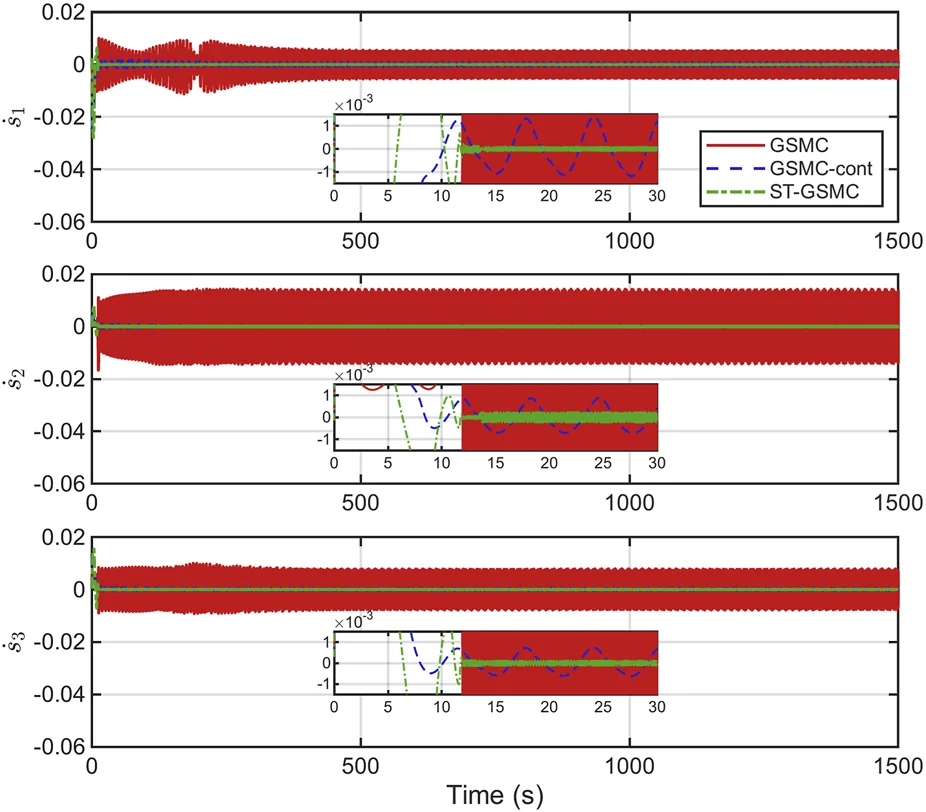
Numerical time derivative of the sliding-variable components. The insets highlight the reaching phase and the different levels of residual high-frequency activity produced by the three controllers.

Finally, Figure 6 shows the virtual input generated by the proposed ST-GSMC, its numerical derivative, and the equivalent-control residual

$$w\left(t\right)=v\left(t\right)+d\left(t\right),\;d\left(t\right)=J^{-1}d_{\tau }\left(t\right),$$

where $d_{\tau }$ is the physical disturbance torque and d is the corresponding disturbance in the normalized angular dynamics. According to the equivalent-control condition derived in Section 4, once the sliding manifold is reached, w(t) converges to zero and, consequently, v(t) approaches −d(t) .

**FIGURE 6:**
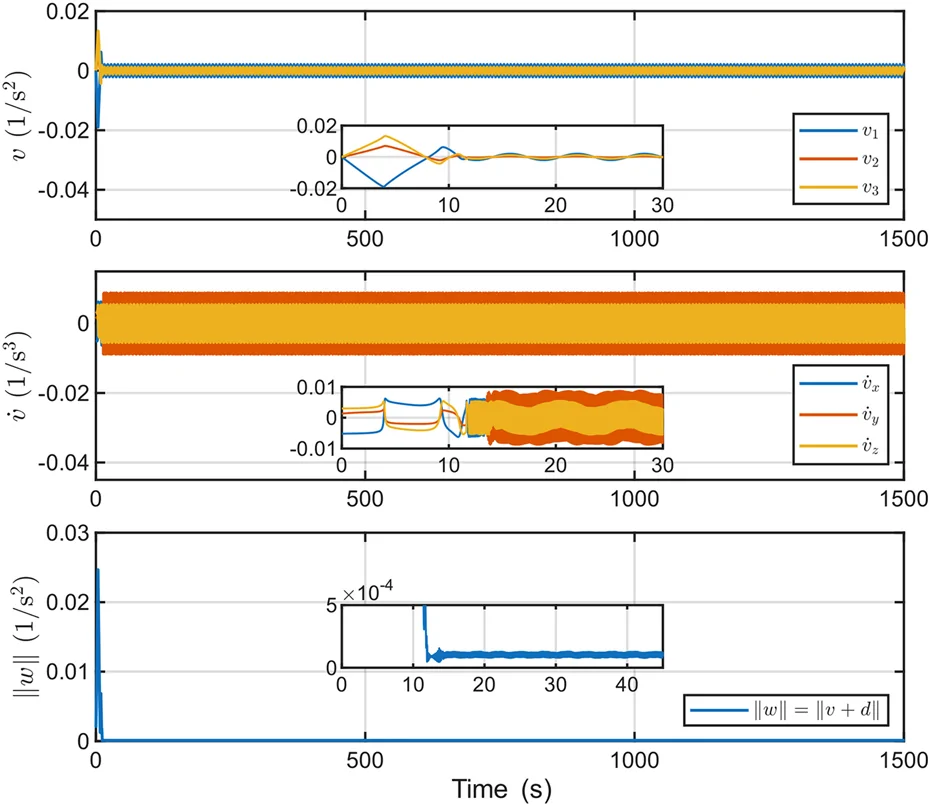
Virtual-input components, their numerical time derivatives, and equivalent-control residual for the proposed ST-GSMC. The upper and middle panels show v and $\dot{v}$ , respectively, while the lower panel reports ‖w‖=‖v+d‖ . The insets magnify the initial reaching transient and the bounded residual oscillations associated with the sampled-data implementation.

The first panel shows that, after the initial reaching transient, the components of the virtual input settle into bounded periodic signals consistent with the frequency of the applied sinusoidal disturbance. The second panel confirms that the numerical derivative of the virtual input remains bounded, although a small high-frequency ripple is visible because of the discrete implementation. More importantly, the third panel directly shows the rapid reduction of ‖w(t)‖ . After the transient, the residual remains in a small oscillatory neighborhood of zero. This practical convergence confirms that the virtual input compensates the matched generalized disturbance, consistently with the theoretical result v→−d .

To complement the qualitative comparison provided by the time histories, Table 3 reports steady-state tracking and oscillation metrics for the three controllers. All quantities are evaluated over t∈[1200,1500] s, thereby excluding the initial reaching transient. In addition to the RMSE values, the table reports the maximum attitude error and the largest component-wise peak-to-peak (P2P) oscillations of the angular-velocity error and sliding variable. For ST-GSMC, the equivalent-control residual w=v+d is additionally quantified through its steady-state RMS norm.

**TABLE 3:** Steady-state tracking and oscillation metrics for the three controllers. All metrics are evaluated over t∈[1200,1500] s.

Controller	${RMSE}_{\theta }$ (deg)	${Max}_{\theta }$ (deg)	${RMSE}_{\left\|e_{\omega }\right\|}$ (rad/s)	${P2P}_{e_{\omega }}$ (rad/s)	${RMSE}_{\left\|s\right\|}$ (rad/s)	${P2P}_{s}$ (rad/s)	${RMS}_{\left\|w\right\|}$ ( ${\text{s}}^{-2}$ )
GSMC	$1.970\times 10^{-3}$	$2.948\times 10^{-3}$	$1.170\times 10^{-4}$	$2.901\times 10^{-4}$	$1.170\times 10^{-4}$	$2.900\times 10^{-4}$	–
GSMC-cont	$8.945\times 10^{-2}$	$1.251\times 10^{-1}$	$1.571\times 10^{-3}$	$3.894\times 10^{-3}$	$1.571\times 10^{-3}$	$3.898\times 10^{-3}$	–
ST-GSMC	$3.117\times 10^{-4}$	$8.251\times 10^{-4}$	$2.839\times 10^{-6}$	$7.146\times 10^{-6}$	$2.838\times 10^{-6}$	$7.134\times 10^{-6}$	$9.534\times 10^{-5}$

Under the considered numerical conditions, ST-GSMC exhibits the smallest steady-state errors and residual oscillations among the three controllers. In particular, relative to the discontinuous GSMC benchmark, the angular-velocity RMSE and its largest component-wise P2P oscillation are both reduced by approximately a factor of 41, with a comparable reduction observed for the sliding variable. These quantitative values are consistent with the reduced residual activity visible in Figures 2–5.

### 5.3 Monte Carlo robustness and gain-sensitivity campaigns

Two complementary numerical campaigns are conducted. The first is a fixed-gain Monte Carlo robustness study that evaluates the controller under variations in the initial conditions, inertia properties, matched disturbances, actuator-gain errors, and actuator noise. The second is a one-at-a-time gain-sensitivity study in which either $k_{1}$ or $k_{2}$ is varied, while the plant parameters, initial condition, disturbance realization, actuator uncertainty, and the disturbance are held fixed.

The complete data set comprises 700 long-duration simulations: $N_{\text{rob}}=400$ robustness trials and $N_{\text{gain}}=300$ gain-sensitivity trials, with 150 simulations assigned to each of the $k_{1}$ and $k_{2}$ sweeps. In both campaigns, the plant is propagated over T=500 s using an integration step of Δt=0.01 s, while the controller is updated at 10 Hz. Between controller updates, the commanded torque is maintained by a zero-order hold.

Both campaigns use λ=0.075 , whereas the controller comparison presented in Section 5.2 uses λ=0.01 . The larger value adopted in the Monte Carlo campaigns accelerates the reduced-order dynamics, shortens the maneuver duration, and keeps the total computational cost within practical limits. Accordingly, the results of the Monte Carlo campaigns and those of Section 5.2 should not be interpreted as a direct quantitative comparison under identical controller tuning. Rather, they represent complementary evaluations performed under different numerical and computational settings.

The controller is implemented using the nominal inertia matrix reported in Table 2. The plant, however, uses a trial-dependent inertia matrix generated according to the prescribed uncertainty ranges. To increase the variability and dynamic significance of the inertia uncertainty, the nominal inertia matrix used as the basis of the Monte Carlo plant model is amplified by a factor of ten. The reference trajectory is the same as that employed in Section 5.2.

The actuator model used in the Monte Carlo campaigns differs from that adopted in the controller comparison. In the campaigns, the actuator is instead represented by a lumped body-torque model with component-wise saturation, multiplicative gain uncertainty, and additive noise. This choice prevents actuator saturation from dominating the robustness and gain-sensitivity assessment over the prescribed ranges of $k_{1}$ and $k_{2}$ .

Moreover, because the Monte Carlo plant inertia is increased by a factor of ten and the larger value of λ produces a faster desired closed-loop maneuver, a comparatively high body-torque saturation limit is helpful. This choice prevents actuator saturation from dominating the gain-sensitivity results and permits a sufficiently broad range of $k_{1}$ and $k_{2}$ values to be investigated.

#### 5.3.1 Fixed-gain robustness campaign

The controller gains are fixed at

$$\lambda =0.075,\;k_{1}=0.1,\;k_{2}=0.005.$$ (79)

The randomized quantities are summarized in Table 4. Initial attitudes are drawn uniformly at random over SO(3) . The initial angular-rate tracking error is sampled uniformly in a three-dimensional ball of radius 0.15 rad/s. Physically admissible inertia matrices are generated by perturbing the principal moments and principal axes of the nominal inertia. Candidate principal moments are sampled using multiplicative factors in [0.2, 1.8] and are accepted only when the rigid-body triangle inequalities are satisfied. The principal-axis frame is independently perturbed by a bounded rotation of at most 10° .

**TABLE 4:** Randomized quantities in the fixed-gain robustness campaign.

Category	Quantity	Sampling rule / range
Initial condition	Relative attitude $R_{d}{\left(0\right)}^{T}R\left(0\right)$	Uniform on SO(3)
	Initial rate error $\omega_{e}\left(0\right)$	Uniform in $\left\{\omega \in R^{3}:\|\omega {\|}_{2}\leq 0.15\right\}$ rad/s
Plant inertia	Principal moments	Multiplicative factors proposed uniformly in [0.2, 1.8]
	Principal-axis mismatch	Isotropic bounded rotation with angle not exceeding 10°
Matched disturbance	Bias $d_{0}$	Uniform in a ball of radius $10^{-3}$ N ⋅ m
	Sinusoidal amplitude a	Uniform in a ball of radius $10^{-3}$ N ⋅ m
	Period and phase	Period independently uniform in [40, 250] s; phase uniform in [0,2π]
Actuator	Multiplicative disturbance gain Γ	Independent diagonal entries uniform in [0.85, 1.15]
	Noise standard deviation	Uniform in $\left[0,10^{-3}\right]$ N ⋅ m; zero-mean Gaussian samples at every plant step
	Torque limit	Component-wise saturation at ±0.5 N ⋅ m

The matched disturbance is smooth and differentiable:

$$d_{\tau }\left(t\right)=d_{\tau 0}+a⊙\sin\left(\varpi t+\phi \right),$$ (80)

where ⊙ denotes component-wise multiplication. Both $d_{0}$ and a are sampled in balls of radius $10^{-3}$ N ⋅ m, the component periods belong to [40, 250] s, and the phases are uniformly distributed over [0,2π] .

The Lyapunov feasibility of the fixed controller gains is also checked for the smooth matched-disturbance component of the robustness campaign. Since

$$\|a\|\leq 10^{-3}\;N\;m,\;T_{i}\geq 40\;s,$$

the physical disturbance-torque derivative satisfies

$$\|{\dot{d}}_{\tau }\left(t\right)\|\leq 10^{-3}\frac{2\pi }{40}.$$

Using the nominal inertia matrix, this gives the ordinary generalized disturbance-rate bound

$$\Delta_{d}\leq \frac{10^{-3}\left(2\pi /40\right)}{\sqrt{\lambda_{\min}\left(J\right)}}=2.2214\times 10^{-4}.$$

For the fixed gains Equation 79 the Lyapunov parameters

$$\alpha_{1}=0.2,\;\alpha_{2}=30,\;\alpha_{12}=-1$$

give

$$\alpha_{1}\alpha_{2}-\alpha_{12}^{2}=5>0,\;\lambda_{\min}\left(P\right)=0.1665,$$

and

$$\lambda_{\min}\left(Q-R_{d}\right)=9.5109\times 10^{-3}>0.$$

The largest disturbance-rate value for which the numerical feasibility problem remains solvable with these gains is

$$\Delta_{d,crit}=3.0902\times 10^{-3},$$

which is approximately 13.9 times the adopted ordinary-derivative bound. The smooth matched-disturbance component therefore satisfies the matrix condition $Q-R_{d}≻0$ with a strict feasibility margin. This Lyapunov verification concerns the smooth matched-disturbance component under the nominal inertia metric.

The applied body torque is

$$\tau_{a}={\text{sat }}_{\bar{\tau }}\left(\Gamma \tau_{c}+\eta \right),\;\bar{\tau }={\left[\begin{array}{ccc} 0.5 & 0.5 & 0.5 \end{array}\right]}^{T}N\;m,$$ (81)

where Γ is diagonal with entries in [0.85, 1.15]. For each trial, the standard deviation of the zero-mean Gaussian actuator noise η is sampled uniformly in $\left[0,10^{-3}\right]$ N ⋅ m and is then held fixed for that trial.

#### 5.3.2 Gain-sensitivity campaign

The gain-sensitivity campaign uses a single reproducible off-nominal scenario and common random numbers. Hence, every candidate gain is exposed to exactly the same plant, initial condition, disturbance, actuator uncertainty, and actuator-noise realization. The parameter λ remains fixed at its nominal value, while $k_{1}$ and $k_{2}$ are varied separately using homogeneous sampling:

$$k_{1}\in \left[0.02,0.30\right],\;k_{2}\in \left[0.001,0.015\right].$$ (82)

The fixed off-nominal scenario employs an initial attitude error of 160° , the initial rate error ${\left[0.08,-0.05,0.04\right]}^{T}$ rad/s, a perturbed physical inertia, a smooth matched disturbance, the actuator-gain matrix Γ=diag (1.08,0.94,1.05) , and actuator-noise standard deviation $5\times 10^{-4}$ N ⋅ m. The two gain sweeps and their fixed parameters are summarized in Table 5.

**TABLE 5:** Gain-sensitivity campaign.

Sweep	Range	Fixed gains	Trials
$k_{1}$	[0.02, 0.30]	λ=0.075 , $k_{2}=0.005$	150
$k_{2}$	[0.001, 0.015]	λ=0.075 , $k_{1}=0.1$	150

Before performing the gain-sensitivity simulations, each sampled positive gain pair $\left(k_{1},k_{2}\right)$ is evaluated with the same approach introduced in Section 4.1. The auxiliary Lyapunov parameters are generated according to (47), which guarantees P≻0 and Q≻0 for every $k_{1}>0$ and $k_{2}>0$ .

For numerical screening, the normalized gain-ratio indicator

$$c_{⋆}\left(k_{1},k_{2}\right)≜\frac{8k_{2}}{k_{1}^{2}+8k_{2}}\in \left(0,1\right)$$ (83)

is evaluated for every candidate. For the certificate Equation 47, it is related to the determinant condition of P through

$$\alpha_{1}\alpha_{2}-\alpha_{12}^{2}=\frac{1}{1-c_{⋆}}.$$ (84)

Only candidates satisfying the campaign-specific threshold

$$c_{⋆}>c_{\text{safety}},\;c_{\text{safety}}=0.15,$$ (85)

are retained.

This threshold is an additional numerical gain-balance criterion used to exclude combinations for which $k_{1}^{2}$ is excessively large relative to $k_{2}$ . It is not required by the nominal finite-time stability theorem, since nominal Lyapunov feasibility is already guaranteed by the construction in Section 4.1.

The screening therefore verifies nominal continuous-time Lyapunov feasibility together with the additional campaign-specific gain-balance criterion. The trial-dependent robust condition $Q-R_{d}≻0$ is not enforced for every sampled gain pair, and the screening does not include the effects of inertia mismatch, actuator uncertainty, saturation, noise, or sampled-data implementation. Accordingly, the gain-sensitivity campaign is interpreted as an empirical evaluation of nominally certified gain pairs under a common prescribed off-nominal scenario, rather than as a numerical verification of the full robust finite-time conditions for every candidate.

#### 5.3.3 Performance metrics

Two distinct settling quantities are retained. The reaching time $t_{r}$ characterizes convergence to a practical neighborhood of the second-order sliding set, whereas the maneuver settling time $t_{m}$ characterizes complete attitude and angular-rate tracking. Specifically,

$$\|s\left(t\right)\|<\varepsilon_{s},\;\varepsilon_{s}=6.31\times 10^{-4}\;rad/s,$$

is used for $t_{r}$ . The sliding threshold is selected consistently with the selected maneuver tolerances. Since

$$s=\omega_{e}+\lambda \text{ Log }{\left(R_{e}\right)}^{\vee }$$

and

$$\left\|\text{Log }{\left(R_{e}\right)}^{\vee }\right\|=\theta_{e},$$

the triangle inequality gives

$$\|s\|\leq \|\omega_{e}\|+\lambda \theta_{e}.$$

Accordingly, the practical sliding threshold is chosen as

$$\varepsilon_{s}=\varepsilon_{\omega }+\lambda \varepsilon_{\theta }=5\times 10^{-4}+0.075\text{ deg}2\text{rad }\left(0.1\right)=6.31\times 10^{-4}\;rad/s.$$

For the maneuver settling time $t_{m}$ , the conditions

$$\theta_{e}\left(t\right)<0.1{}^{\circ},\;\|\omega_{e}\left(t\right)\|<5\times 10^{-4}\;rad/s$$

are used. For either settling metric, a candidate time is accepted only if the relevant condition holds continuously for a 15 s dwell interval and remains satisfied for at least 98% of the samples over the remaining simulation horizon. The first accepted sample defines the corresponding settling time. If no sample passes both tests, the trial is marked unsuccessful for that metric. The steady-state attitude and angular-rate RMSEs are evaluated over $\left[t_{m},T\right]$ , not over $\left[t_{r},T\right]$ . The control-effort metric is computed using the applied torque after gain uncertainty, actuator noise, and saturation:

$$E_{\tau }=\int_{0}^{T}\|\tau_{a}\left(t\right)\|\;dt.$$

The peak-torque metric is

$$\tau_{\text{pk}}=\underset{t\in \left[0,T\right]}{\max}\|\tau_{a}\left(t\right)\|.$$

The complete set of performance metrics is summarized in Table 6.

**TABLE 6:** Monte Carlo performance metrics.

Metric	Definition
$t_{r}$	Sliding reaching time based on $\|s{\|}_{2}<\varepsilon_{s}$
$t_{m}$	Maneuver settling time based jointly on attitude and angular-rate errors
${RMSE}_{\theta ,ss}$	$\sqrt{\frac{1}{T-t_{m}}\int_{t_{m}}^{T}\theta_{e}{\left(t\right)}^{2}dt}$
${RMSE}_{\omega ,ss}$	$\sqrt{\frac{1}{T-t_{m}}\int_{t_{m}}^{T}\|\omega_{e}\left(t\right){\|}_{2}^{2}dt}$
$E_{\tau }$	Applied control effort $\int_{0}^{T}\|\tau_{a}\left(t\right){\|}_{2}dt$
$\tau_{\text{pk}}$	Maximum applied torque norm $\max_{t}\|\tau_{a}\left(t\right){\|}_{2}$

#### 5.3.4 Robustness results

All 400 fixed-gain trials reached the practical sliding neighborhood and satisfied the maneuver-settling criterion, yielding a 100% success rate for both metrics within the considered uncertainty envelope. Table 7 reports the median, 95th percentile, and worst observed value. The corresponding empirical distributions are shown in Figure 7.

**TABLE 7:** Aggregate results of the fixed-gain robustness campaign $\left(N_{\text{rob}}=400\right)$ .

Metric	Median	95th percentile	Worst
Reaching time $t_{r}$ [s]	18.95	30.91	38.90
Maneuver settling time $t_{m}$ [s]	97.74	102.82	104.96
${RMSE}_{\theta ,ss}$ [deg]	$1.294\times 10^{-2}$	$1.486\times 10^{-2}$	$2.556\times 10^{-2}$
${RMSE}_{\omega ,ss}$ [rad/s]	$2.289\times 10^{-5}$	$3.508\times 10^{-5}$	$4.698\times 10^{-5}$
Control effort $E_{\tau }$ [N ⋅ m ⋅ s]	5.647	9.333	13.100
Peak torque norm $\tau_{\text{pk}}$ [N ⋅ m]	0.297	0.445	0.516

**FIGURE 7:**
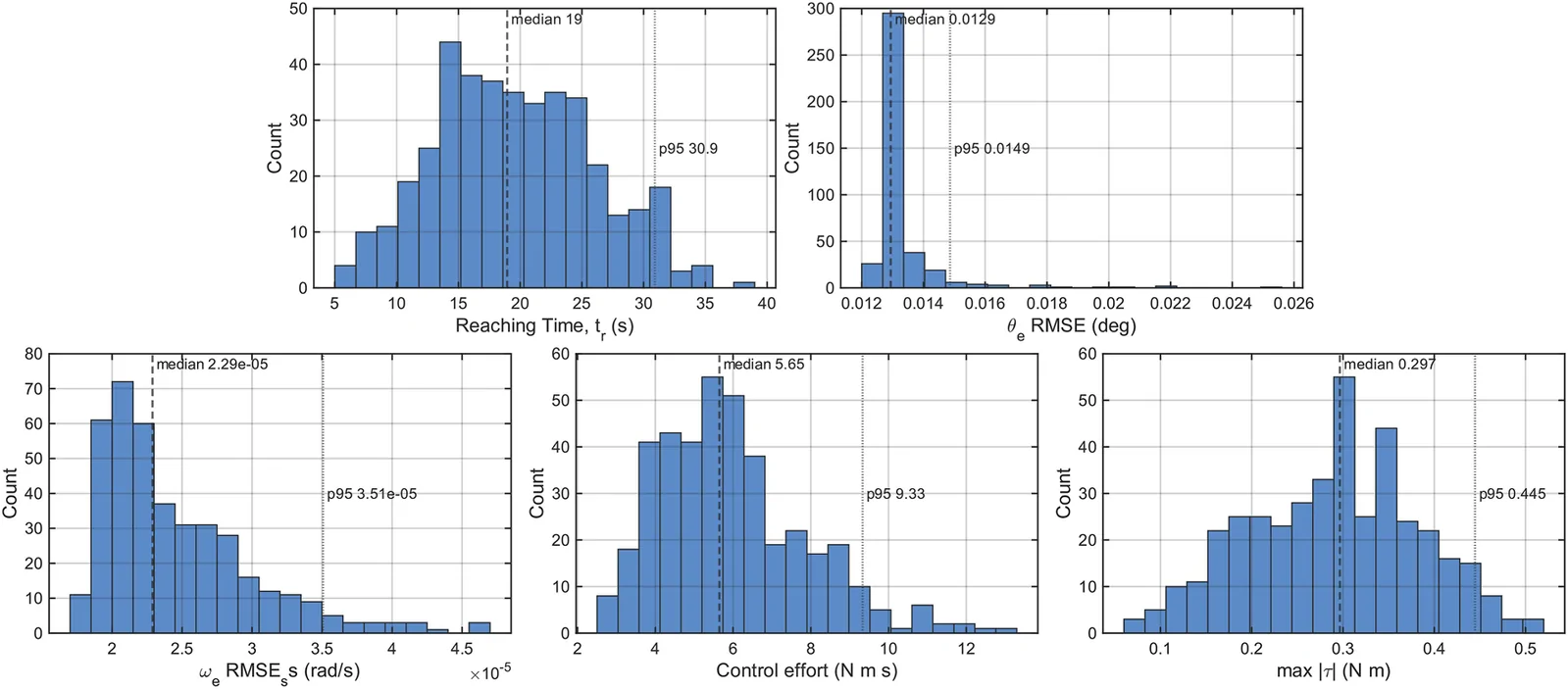
Distributions obtained in the fixed-gain robustness campaign. Dashed and dotted vertical lines indicate the median and 95th percentile, respectively.

The median reaching time is 18.95 s and the 95th percentile is 30.91 s. In contrast, the median complete maneuver-settling time is 97.74 s, confirming that finite-time reaching of the sliding neighborhood precedes complete attitude tracking. The median steady-state pointing RMSE is $1.294\times 10^{-2}$ deg, while the median angular-rate RMSE is $2.289\times 10^{-5}$ rad/s. No component-wise torque-saturation event occurs in the fixed-gain robustness campaign.

#### 5.3.5 Gain-sensitivity results

These results also provide the numerical basis for the practical gain-selection guidance discussed in Section 4.2. All gain-sensitivity trials satisfy both the sliding-reaching and maneuver-settling criteria. The banded statistics are summarized in Table 8. Figure 8 shows that $k_{1}$ has the dominant effect on reaching speed: increasing $k_{1}$ reduces $t_{r}$ almost monotonically over the tested range. This improvement is accompanied by increasing rate RMSE, control effort, and peak torque at the upper end of the range. In particular, the median reaching time decreases from 75.75 s in the low- $k_{1}$ band to 13.30 s in the high- $k_{1}$ band, whereas the median control effort increases from 7.65 to 9.23 N ⋅ m ⋅ s after attaining a minimum in the middle band.

**TABLE 8:** Gain-sensitivity statistics in three equal-count gain bands. Each row contains 50 trials.

Gain	Band	Sampled range	Median $t_{r}$ [s]	Median ${RMSE}_{\theta ,ss}$ [deg]	Median $E_{\tau }$ [N ⋅ m ⋅ s]	Median $\tau_{\text{pk}}$ [N ⋅ m]
$k_{1}$	Low	[0.0202, 0.0485]	75.75	0.01271	7.65	0.202
	Middle	[0.0495, 0.1209]	29.80	0.01299	6.38	0.267
	High	[0.1223, 0.2995]	13.30	0.01306	9.23	0.556
$k_{2}$	Low	[0.00101, 0.00246]	24.80	0.01302	5.96	0.322
	Middle	[0.00248, 0.00603]	23.40	0.01300	6.53	0.322
	High	[0.00617, 0.01481]	23.40	0.01304	7.67	0.324

**FIGURE 8:**
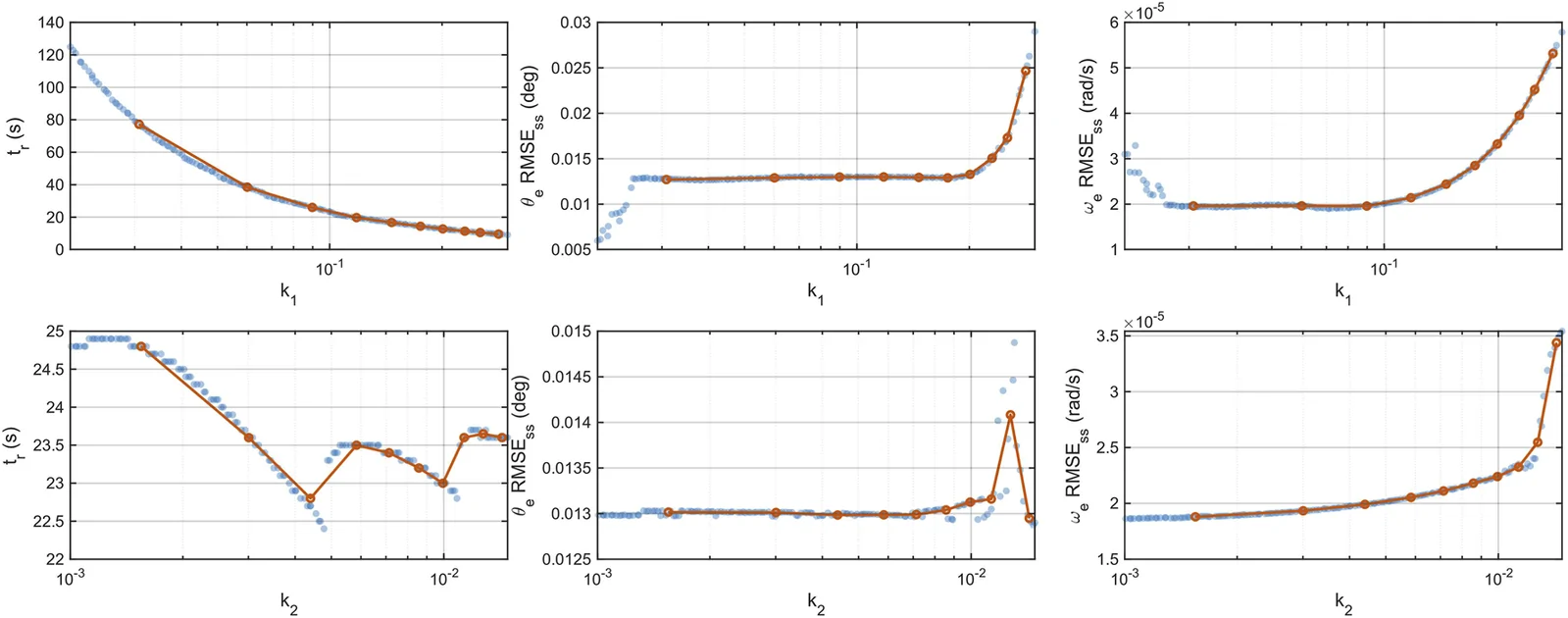
One-at-a-time sensitivity of the reaching time and steady-state tracking errors to $k_{1}$ (top row) and $k_{2}$ (bottom row), with λ=0.075 . Blue markers denote individual trials and the orange curves denote binned medians.

The influence of $k_{2}$ on reaching time is comparatively weak over the tested interval: the median $t_{r}$ changes from 24.80 s in the low band to 23.40 s in the high band. Increasing $k_{2}$ nevertheless produces a gradual increase in control effort and angular-rate RMSE. The steady-state attitude RMSE remains nearly constant over most of both sweeps and deteriorates only near the largest tested gains. These trends indicate a clear trade-off between rapid reaching and actuation demand, particularly for $k_{1}$ . The associated control-effort and peak-torque trends are reported in Figure 9. The proportional increase in the angular velocity tracking error with respect to the gains is consistent with sampled implementation and residual high-frequency activity.

**FIGURE 9:**
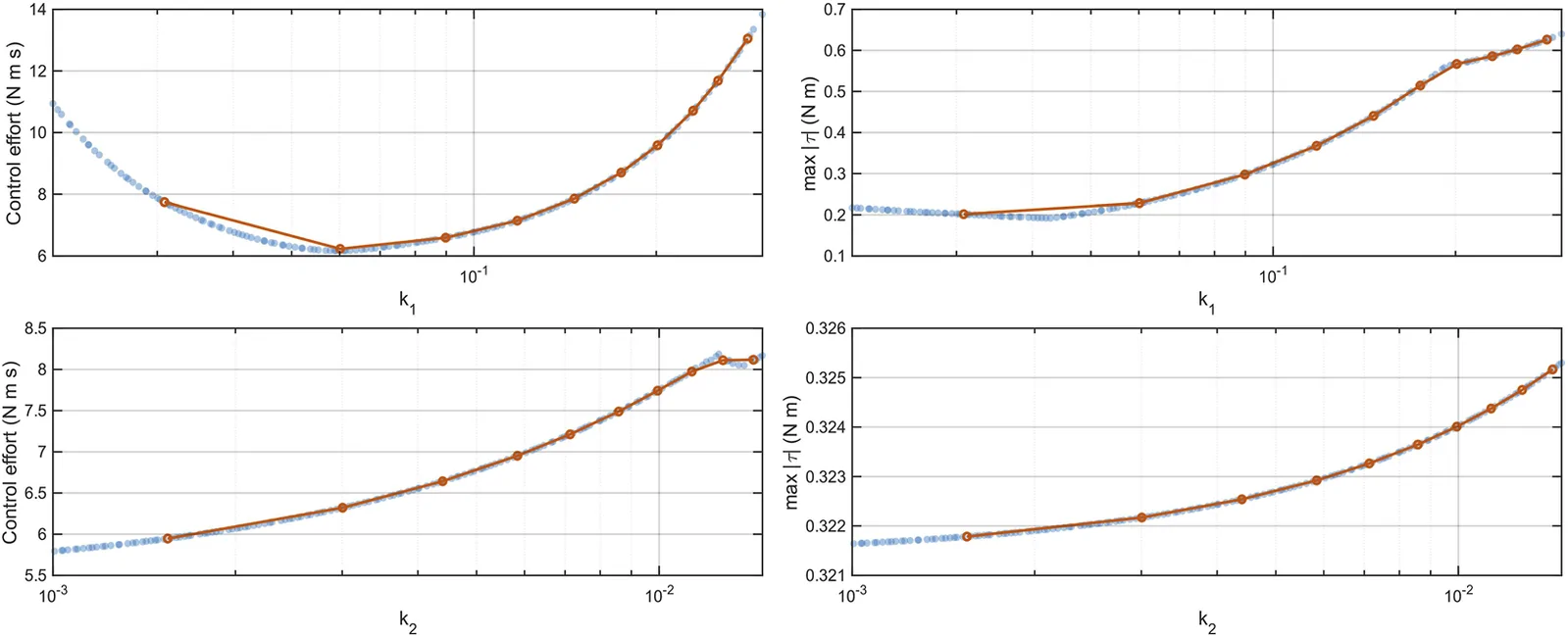
One-at-a-time sensitivity of applied control effort and peak torque norm to $k_{1}$ (top row) and $k_{2}$ (bottom row), with λ=0.075 .

## 6 Conclusion

This paper developed an intrinsic multivariable super-twisting reaching law for fully actuated mechanical systems evolving on Lie groups. Building on the existing state-space Lie-group and sliding-subgroup construction, both layers of the proposed reaching mechanism are formulated through covariant derivatives on the Lie algebra. A strict Lyapunov analysis established finite-time convergence to the second-order sliding set and provided constructive nominal and robust gain conditions together with explicit reaching-time bounds. For sufficiently regular matched disturbances, the analysis further established finite-time convergence of the internal super-twisting state to the equivalent disturbance-compensation action. Once the sliding subgroup is reached, the inherited reduced dynamics ensure almost-global asymptotic convergence of the complete tracking error.

The general framework was specialized to spacecraft attitude tracking on SO(3) and assessed through comparative, robustness, and gain-sensitivity simulations. These numerical results support the finite-time reaching, disturbance-rejection, and tracking behavior predicted by the continuous-time analysis, while also illustrating the reduced residual oscillatory activity obtained with the proposed higher-order reaching mechanism under sampled-data implementation. Within the considered sampled-data scenario, ST-GSMC also exhibits lower steady-state tracking errors and residual oscillation levels than the two first-order geometric sliding-mode baselines, consistently with the quantitative metrics reported in Table 3.

The finite-time reaching and exact matched-disturbance compensation results are continuous-time theoretical guarantees obtained under the stated full-actuation, matching, and disturbance-regularity assumptions. The sampled-data simulations instead demonstrate practical convergence to small residual neighborhoods and reduced oscillatory activity; they should not be interpreted as implying complete elimination of discretization-induced chattering or as extending the theoretical guarantees beyond the adopted assumptions. Moreover, the SO(3) specialization retains the usual topological limitation associated with the logarithmic attitude error, so that the resulting tracking property is almost global rather than global. These aspects motivate future extensions toward underactuated and unmatched-disturbance settings and validation within more realistic spacecraft-control environments.

## Data Availability

The raw data supporting the conclusions of this article will be made available by the authors, without undue reservation.
