# Supplementary material for: Intrinsic geometric super-twisting control on Lie groups with application to spacecraft attitude tracking
Source: Front Robot AI. 2026 Oct 28;13:1961299. doi: 10.3389/frobt.2026.1961299 (PMC13751411; doi:10.3389/frobt.2026.1961299)
Supplement: Supplementary file 1 [file Supplementaryfile1.pdf]

Supplementary Material

1 SUPPLEMENTARY APPENDIX

1.1 Explicit $\mathrm{SO}(3)$ specialization and benchmark controllers

For the inertia metric $\mathbb{I}(x,y) = x^T J y$, the Levi–Civita connection on $\mathfrak{so}(3) \simeq \mathbb{R}^3$ is

$$\nabla_x^{\mathfrak{so}(3)} y = \frac{1}{2} x \times y - \frac{1}{2} J^{-1} \left[(Jy) \times x + (Jx) \times y \right]. \tag{S1}$$

Accordingly, the virtual-state dynamics in Eq. (73) of the main article can be written explicitly as

$$\begin{aligned} \dot{v} \in & -k_2 \operatorname{Sgn}_J(s) - \frac{1}{2} s \times v \\ & + \frac{1}{2} J^{-1} \left[(Jv) \times s + (Js) \times v \right], \end{aligned} \tag{S2}$$

where

$$\operatorname{Sgn}_J(s) = \begin{cases} \dfrac{s}{\sqrt{s^T J s}}, & s \neq 0, \\ \{ z \in \mathbb{R}^3 : z^T J z \leq 1 \}, & s = 0. \end{cases} \tag{S3}$$

Substitution of (S1) and the attitude-error relations Eqs. (65)–(72) of the main article into the generalized control law Eq. (24) of the main article yields the spacecraft torque Eq. (74) of the main article. Substitution of that torque into Eq. (62) of the main article gives

$$\nabla_s s = -k_1 (s^T J s)^{-1/4} s + v + J^{-1} d_\tau, \tag{S4}$$

which recovers the intrinsic reaching dynamics with $d = J^{-1} d_\tau$.

For completeness, the two first-order controllers used in Section 5.2 of the main article can be represented by the common torque law

$$\begin{aligned} \tau_q = & (J\Omega) \times (\lambda \Omega_u(R_e) - \sigma) \\ & + J \left[\dot{\sigma} - \lambda \dot{\Omega}_u(R_e) - k_s \Psi_q(s) \right], \end{aligned} \tag{S5}$$

with

$$\Psi_q(s) = \begin{cases} (s^T J s)^p s + \operatorname{Sgn}_J(s), & \textit{GSMC}, \\ \left[(s^T J s)^p + (s^T J s)^{-q_c} \right] s, & \textit{GSMC-cont}, \end{cases} \qquad 0 < q_c < \frac{1}{2}. \tag{S6}$$

The first case corresponds to the discontinuous choice $q = 1/2$, whereas the second replaces the metric-sign contribution with a continuous power-law term.
